# Antimicrobial Resistance in Aquaculture: Risk Mitigation within the One Health Context

**DOI:** 10.3390/foods13152448

**Published:** 2024-08-02

**Authors:** Milan Milijasevic, Slavica Veskovic-Moracanin, Jelena Babic Milijasevic, Jelena Petrovic, Ivan Nastasijevic

**Affiliations:** 1 Institute of Meat Hygiene and Technology, 11000 Belgrade, Serbia; milan.milijasevic@inmes.rs (M.M.); slavica.veskovic@inmes.rs (S.V.-M.); jelena.babic@inmes.rs (J.B.M.); 2 Scientific Veterinary Institute ‘Novi Sad’, 21113 Novi Sad, Serbia; jelena@niv.ns.ac.rs

**Keywords:** antimicrobial resistance, aquaculture, fisheries, risk mitigation, One Health

## Abstract

The application of antimicrobials in aquaculture primarily aims to prevent and treat bacterial infections in fish, but their inappropriate use may result in the emergence of zoonotic antibiotic-resistant bacteria and the subsequent transmission of resistant strains to humans via food consumption. The aquatic environment serves as a potential reservoir for resistant bacteria, providing an ideal breeding ground for development of antimicrobial resistance (AMR). The mutual inter-connection of intensive fish-farming systems with terrestrial environments, the food processing industry and human population creates pathways for the transmission of resistant bacteria, exacerbating the problem further. The aim of this study was to provide an overview of the most effective and available risk mitigation strategies to tackle AMR in aquaculture, based on the One Health (OH) concept. The stringent antimicrobial use guidelines, promoting disease control methods like enhanced farm biosecurity measures and vaccinations, alternatives to antibiotics (ABs) (prebiotics, probiotics, immunostimulants, essential oils (EOs), peptides and phage therapy), feeding practices, genetics, monitoring water quality, and improving wastewater treatment, rather than applying excessive use of antimicrobials, can effectively prevent the development of AMR and release of resistant bacteria into the environment and food. The contribution of the environment to AMR development traditionally receives less attention, and, therefore, environmental aspects should be included more prominently in OH efforts to predict, detect and prevent the risks to health. This is of particular importance for low and middle-income countries with a lack of integration of the national AMR action plans (NAPs) with the aquaculture-producing environment. Integrated control of AMR in fisheries based on the OH approach can contribute to substantial decrease in resistance, and such is the case in Asia, where in aquaculture, the percentage of antimicrobial compounds with resistance exceeding 50% (P50) decreased from 52% to 22% within the period of the previous two decades.

## 1. Introduction

Over decades, human activities in the agri-food chain have significantly affected the environment, resulting in depletion of natural resources and biodiversity. All forms of agricultural production represent ideal platforms for development and expansion of AMR through diverse ecosystems [1]. AMR occurs when bacteria, viruses, fungi and parasites no longer respond in vivo to antimicrobials in a prescribed dosage and, as a result of drug resistance, infections become difficult or impossible to treat, increasing the risk of disease spread, severe illness, disability and death [2].

In essence, AMR represents a microorganism’s capacity to withstand the growth-inhibitory or lethal activity of an antimicrobial, beyond the typical susceptibility of the specific bacterial species [3]. It is the result of interactions between the microbial cell, its environment and the antimicrobial agent [1]. When people or animals come into contact with resistant pathogens, they may be subjected to infections that are no longer responsive to the available antimicrobial treatment. This not only results in the worsening of patients’ health and potential fatalities, but also leads to an increase in healthcare expenses due to prolonged hospitalization, disabilities and the ongoing spread of diseases. Therefore, an indiscriminate use of antimicrobials across veterinary, agricultural, and medical sectors is a significant contributing factor to the development of AMR [1]. The World Health Organization (WHO) raised concerns about the alarming levels AMR in various parts of the world [2]. In a study on global burden of AMR using different bacterial pathogen-drug combinations it was observed that 4.95 million AMR-associated deaths occurred in 2019 and the six leading pathogens for deaths associated with AMR were identified: *Escherichia coli*, *Staphylococcus aureus*, *Klebsiella pneumoniae*, *Streptococcus pneumoniae*, *Acinetobacter baumannii*, and *Pseudomonas aeruginosa* [4]. Further, the AMR is responsible for an annual toll of 25,000 deaths in the European Union (EU) [5] of which 72.4% has been attributed to the health-care-system nosocomial infections [6]. It is estimated that by 2050, bacterial resistance to antimicrobials could increase the annual rate to 10 million deaths and costs and losses to USD 100 trillion [1,7,8,9]. The resistance to fluoroquinolones and β-lactam AB (i.e., carbapenems, cephalosporins, and penicillins) was observed to be the most important, since it was related to more than 70% of AMR-associated deaths [4]. Beyond its substantial public health impact, AMR inflicts significant economic losses, stemming from heightened treatment expenses and reduced productivity due to disease outbreaks. In the EU and European Economic Area (EEA), AMR-associated diseases are responsible for the average disability-adjusted life years (DALYs) at the level of 290.0 per 100,000 population [6]. Annual losses due to treatment and decreased productivity reach EUR 1.5 billion, with the prediction that by the middle of the 21st century, the economic losses caused by AMR will be on par with the recession experienced in 2008 [10].

Parallel to that finding, the study on AMR in aquaculture analyzing existence, design and implementation of NAP revealed that *Vibrio* spp. (resistance genes *hflk* and *chiA*) [11], *Aeromonas* spp. (resistant genes *tetE* and *tetA*) [12], *Streptococcus* (resistance genes *tet*(O), *tet*(M), *erm*(B)), [13] and *Edwardsiella* (resistance genes *bla*TEM, *sul*1, *tet*A, *bla*CTX-M, *aad*A1, *qnr*S, and *qnr*A) [14] were the most present resistant bacteria in the fish farming sector. The most represented classes of AB associated with AMR in aquaculture were β-lactam AB, tetracyclines, sulfonamides, macrolides, and fluoroquinolones [15,16]. 

The use of antimicrobials, whether warranted or not, has the potential for the development of AMR among microorganisms. It is essential to acknowledge that antimicrobials are frequently employed worldwide for prophylactic purposes and as growth promoters in animal husbandry and agriculture [17]. People and animals regularly exchange strains of AB-resistant bacteria within the ecosystems in which they coexist. The EU introduced a ban on AB as growth promoters in animal feed as of 1 January 2006 [18]. In addition, as of January 2022, the routine use of antimicrobials, either for prophylaxis or treatment of animals is banned in the EU and it only allows the preventative use of antimicrobials, restricted only to individual animals requiring exceptional treatment. Therefore, antimicrobials can also no longer be applied as an alternative to and/or to compensate for the poor practices in livestock farming, such as hygiene and biosecurity [19]. However, outside of Europe many countries still continue to use AB growth promoters to this day [20,21].

While the consumption of antimicrobials in the human health sector of developed countries is steadily declining [22], their utilization in the agricultural sector, particularly in global pig and poultry farming, remains high [23,24] and less-developed countries are witnessing an increase in AB consumption for both medical purposes and animal husbandry. However, the precise data to support this statement are limited due to non-existent systems for monitoring antimicrobial usage in less-developed countries [25]. 

The AMR associated with aquaculture is of global significance since fish, shellfish and other aquatic foods present an important source of animal protein in many regions of the world, either developed (the EU, USA, and Japan) or developing countries (including China, Southeast Asia, India, and Africa). One of the less commonly discussed avenues for AMR transmission is through the consumption of fish meat, especially non-heat-treated ready-to-eat fish meat products, such as sushi and cold-smoked salmon. In order to suppress AMR, it is very important to establish a monitoring system for antimicrobial resistance genes (ARGs) in microorganisms isolated from such fish products.

This review has the goal of providing a comprehensive overview of the current state of knowledge concerning AMR in aquaculture environments. It highlights the most relevant aspects of antimicrobial use in aquaculture, the development and transfer of AMR, detection methods, alternative solutions and practices to antimicrobials, and the most effective risk mitigation strategies to combat AMR within the OH concept. Ultimately, this review aims to deepen our understanding of the complexities surrounding AMR in aquaculture, as something frequently neglected in comparison with other food value chains, and to support the development of evidence-based strategies to address this critical global challenge.

## 2. Materials and Methods

A literature review was performed by identifying and analyzing published articles (research and review scientific papers, technical reports and guidelines by international organizations) published in the domains of public health, zoonotic food-borne pathogens, aquaculture, fish farming and AMR, originating from the scientific databases such as Web of Science, Scopus, Academic Search Complete, IEEE Xplore, PubMed, EBSCO and CAB Abstracts. In addition, the official web sites of selected national AMR monitoring and surveillance schemes were also analyzed. The search algorithm included relevant keywords and phrases related to the topic. A search strategy using defined keywords was based on Boolean operators (AND, OR, NOT) to combine keywords and narrow down results. These included terms like “AMR AND aquaculture”, “AMR AND fish“, “AMR AND fisheries”, “AMR AND fish farming”, “AMR AND foodborne disease”, “AMR and public health”, “AMR AND veterinary medicine“, “AMR AND clinical breakpoints”, “AMR AND epidemiological cut-off values (ECVs)”, “AMR AND detection methods”, “AMR AND alternatives to antimicrobial treatment”, AMR AND risk mitigation”, “AMR AND One Health”, “AMR AND monitoring”, “AMR and drivers”, “AMR and socioeconomic”. The search was carried out for the years between 2000 and 2023. In total, 175 papers, guidelines and technical reports corresponding to the search algorithm have been extracted as a source of evidence-based information for this narrative review. Each source of information was checked by reading through the titles and abstracts of the search results to assess its relevance and eligibility for the given topic. Once a list of relevant articles had been selected, a “snowballing” technique was used to discover more comprehensive and relevant literature by including also the review of the references extracted from the initially searched articles. The selection criteria to identify the relevant articles within the scope of this review were as follows: (1) focus on antimicrobial use (AMU) in aquaculture and associated AMR, (2) risk mitigation strategies to prevent and control AMR in aquaculture, and (3) potential for improvement of inter-sectoral cooperation between environment, veterinary and health authorities within the One Health context to tackle the occurrence of AMR in aquaculture. 

The data, as well as monitoring and surveillance programs on AMR of the major zoonotic foodborne pathogens associated with aquaculture of public-health importance (*Vibrio* spp., *Aeromonas* spp., *Streptococcus*, *Edwardsiella*), indicator/commensal bacteria, and carriers of resistance genes (e.g., *E. coli*, *Enterococcus* spp.), were reviewed, presenting the status in the EU/EEA countries, but also the global importance of AMR in aquaculture.

## 3. Results and Discussion

The most important aspects of AMR in aquaculture, including effective risk mitigation strategies within the OH approach have been presented and recommended as useful guidance for fish farmers and competent authorities. These encompass antimicrobial use in aquaculture, AMR emergence, AMR transfer and dissemination in aquaculture, clinical breakpoints and ECV, detection methods, and alternatives to antimicrobial treatment in aquaculture, as well as a summary of the most effective risk mitigation strategies for AMR in aquaculture and the One Health approach to tackle AMR in aquaculture from the holistic perspective.

Aquaculture is one of the fastest growing sectors of global agricultural production. This agricultural sector produces more than half of the worldʼs seafood, and production has grown globally at 6% per year since 2001 [8]. Currently, aquaculture provides 10% of the protein consumed globally, but that number is expected to grow to 50% by 2030 [10,23]. Aquaculture production is very complex and is under the influence of a large number of ecological, biological, cultural and socioeconomic factors. To reach the expected growth rate, global aquaculture will need to be subjected to a high degree of intensification, which frequently entails the use of antimicrobials (including ABs) for the treatment and prevention of diseases, increasing productivity and compensating for poor biosecurity measures implemented in fish farms [26,27,28]. The majority of aquaculture production occurs in low- and middle-income countries, where the control of the use of antimicrobials and their quality is at a very low level, and where there are many opportunities for people, animals and microorganisms from the environment to come into close contact [23]. AMU can be minimized in aquaculture when farms implement more effective biosecurity measures, provide comprehensive training to workers in husbandry and management practices, and employ various disease prevention measures such as vaccination and improved water quality [8]. Additionally, the use of alternative treatments (e.g., probiotics, immunostimulants, peptide phage therapy, and other natural products), can further reduce the reliance on antimicrobials. Understanding the problem of AMR in aquaculture and defining possible solutions requires a multidisciplinary and holistic approach to address AMR from multiple aspects within the One Health concept reflecting environment–animal–human interconnection.

### 3.1. Antimicrobial Use in Aquaculture

In previous decades, AMU (including ABs) in animals has exceeded the amount used in humans, at the global level [29,30,31,32]. On the other hand, in the EU a decrease in AMU in food animals has been reported, to the extent that it is actually lower than in humans [33]. This is in contrast with other regions worldwide where the AMU is still extensive and its quantity in food-producing animals still exceeds the use in humans [34]. 

Aquaculture stands as one of the fastest-growing food sectors on a global scale, and its rapid expansion entails heightening the risk of disease outbreaks due to the lack of a standardized approach and regulatory framework for disease prevention and treatment practices [35]. Intensive aquaculture, marked by densely stocked fish pools, sub-optimal hygienic conditions, physical stressors (e.g., overcrowding, handling and transportation, aquatic predators, lighting conditions, noise pollution, etc.), and water quality issues (e.g., water pH levels, temperature fluctuations, water flow and aeration) presents a range of challenges [36]. Such intensification, coming alongside more frequent occurrence of aquatic pathogens, led to an increased reliance on antimicrobials, including AB. Consequently, AMR has become a pressing concern, directly affecting a wide array of aquatic species that are farmed, such as catfish, trout, salmon, tilapia and shrimps [37,38,39,40]. The misuse and/or overuse of ABs can also be attributed to misdiagnoses often made by the breeders themselves. In many cases breeders, without veterinary consultation, resorted to AB use in aquaculture to avert widespread mortality due to poor biosecurity and hygienic conditions and to mitigate substantial economic losses [26]. Recently, there have been promising data showing that fish farmers across the world are not only accepting limited antimicrobial/AB usage but also embracing the new reality of industry rules and standards [35]. While AB-free organic fish farming is a foreseeable future, the reality is that the water quality, fish-farm waste, and associated diseases are still ongoing challenges that sometimes require AMU [23,41]. Therefore, it is imperative that effective, safe and cost-competitive solutions are at the disposal as the best alternatives available in place to replace and/or reduce antimicrobial treatments. The overview on research regarding AMR in aquaculture, including classes of antimicrobials that are most frequently used, is given in Table 1.

In aquaculture, antimicrobials are most often administered orally to all fish living in the same pool or cage [53]. The most common route for the delivery of antimicrobials to fish involves blending the AB with specially formulated feed (i.e., medicated feed). Nonetheless, fish are not fully efficient in metabolizing ABs and other medications from their feed, resulting in a significant portion passing through their bodies and being released into the aquatic environment, often in an unaltered form. It has been estimated that approximately 75% of the AB administered to fish are excreted unchanged into the water and are still microbiologically active [53].

A wide range of antimicrobials (including ABs) are in use in aquaculture today. It is the frequent situation that the same classes of ABs are used both for medical purposes and in aquaculture production [40]. The type of antimicrobials, the method of their use, and the frequency and the amount applied depend on a number of factors, such as (i) the species grown in aquaculture, (ii) type of production (e.g., semi-intensive, intensive, pools, cages), (iii) environment, (iv) availability of drugs, (v) the role of veterinary services, (vi) legal frameworks and (vii) market control [27]. For example, quinolones are very often used in aquaculture [40]. Nowadays, several classes of AB banned in the EU are in regular use in aquaculture production in Asia (e.g., China and Vietnam), including chloramphenicol, ciprofloxacin, florfenicol, nitrofurans and enrofloxacin [54,55]. In addition, Carrizo et al. [56] observed the occurrence of amoxicillin, enrofloxacin, moxifloxacin, penicillin G, ciprofloxacin, oxolinic acid, sulfamethoxazole, trimethoprim, penicillin V, doxycycline, flumequine, oxacillin and pipemidic acid in samples of wild salmon in Chile. The presence of erythromycin, azithromycin, roxithromycin, sulfabenzamide, sulfamethazine, sulfapyridine, cephalexin and sulfaguanidine in samples of farmed salmon has been also found in the same study [56].

It is important to note that the afore mentioned classes of AB belong to both VCIA according to the WOAH classification, [50] and HPCIA and CIA in accordance with the WHO [51], with the addition of the newest WHO list of Medically Important Antibiotics (MIAs) providing the categorization of antimicrobials authorized for use in both humans and animals [51].

This is related to the clear evidence of adverse human health consequences that led to increased episodes of treatment failures (including deaths) and increased severity and duration of infections that may occur due to resistant organisms resulting from excessive non-human usage of antimicrobials. To date, there is a scarcity of information and lack of research related to the development and transfer of AMR in fisheries, connecting the AMU in aquaculture and human health.

**Figure 1:**
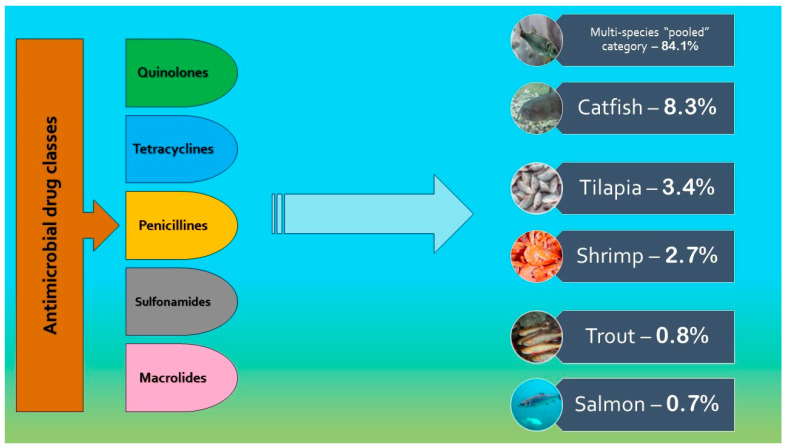
List of antimicrobial drug classes most commonly used in aquaculture and AMU trends (%) by species (adapted from Schar et al. [40]).

Several ABs that are most commonly used in aquaculture are quinolones (27%), tetracyclines (20%), amphenicols (18%) and sulfonamides (14%) (Figure 1) [40]. These ABs are often used to treat or prevent bacterial infections in fish and other aquatic animals, and also belong to the VCIA, emphasizing their importance for public health [50,51]. The most important classes of ABs will be explained in the further sub-paragraphs.

*Quinolones*. Quinolones are a class of synthetic broad-spectrum ABs that are widely used to treat a variety of bacterial infections [40]. The most common quinolones include ciprofloxacin, levofloxacin, moxifloxacin, and ofloxacin. These groups of ABs are considered as HPCIA by the WHO and other health authorities [50,51]. This classification is due to their effectiveness in treating serious and life-threatening infections where few or no alternatives exist. Their broad-spectrum activity makes them essential for managing a wide range of bacterial diseases, particularly in hospital settings. However, the rise in AMR poses a significant public health challenge. As we indicated earlier, resistance to fluoroquinolones is associated with over 70% of deaths linked to AMR [4]. Balancing their benefits against the risks of resistance is essential to maintaining their status as HPCIA.

*Oxytetracycline*. It is a broad-spectrum AB commonly employed in aquaculture to treat bacterial infections in fish, especially those caused by Gram-negative bacteria. Oxytetracycline is poorly absorbed in fish intestines through passive diffusion, necessitating high doses for administration. The pharmacokinetics of oxytetracycline depends on various factors, such as fish species, age, size and health condition, oxytetracycline dosage, water temperature and salinity and the AB’s formulation [57,58]. This results in the slow release of significant AB quantities, increasing selective pressure and potentially fostering the development of oxytetracycline-resistant bacterial strains within the fish’s intestines [53,59]. In fish, oxytetracycline is primarily distributed to the liver, kidneys, and muscle tissues, where it can persist for several days after treatment. Most of the applied oxytetracycline is excreted in feces (70–80%). Although it is easily degraded in seawater, it remains in the sediments for a long time [53]. For example, some studies have shown that the concentration of oxytetracycline in sediments on salmon farms is up to 11 µg/g, while its half-life is from 9 to 415 days [53,60]. This is the likely reason why salmon-farmed marine ecosystems have oxytetracycline-resistant bacteria as high as 25%, compared to less than 5% in non-salmon-farmed locations [53]. Therefore, it is crucial to investigate and regularly monitor the persistence of various ABs in both water and sediments.

*Sulfadiazine*. It is a sulfonamide AB frequently employed in aquaculture for its ability to inhibit bacterial growth and replication. This group is among the most used AB in the European countries, with contributions between 11 and 24% [61]. When bacteria come into contact with sulfadiazine, those possessing resistance genes or mutations that enable them to survive and reproduce will thrive, passing on their resistant traits to subsequent generations. Research indicates that the use of sulfadiazine in aquaculture can indeed contribute to the emergence of sulfonamide-resistant bacteria, affecting both fish and the surrounding aquatic environment. Such resistant bacteria have been detected in fish, and shellfish, as well as in sediment and water samples from aquaculture facilities [62].

*Florfenicol*. It is a relatively new AB, belonging to the class of phenicols, gaining popularity in aquaculture due to its effectiveness against a broad spectrum of bacteria and a lower risk of resistance development. It is frequently used to combat bacterial infections in fish, particularly those caused by Gram-negative bacteria [63]. The impact of florfenicol on AMR is multifaceted and can hinge on various factors, including treatment dosage and duration, frequency of use, and the specific bacteria involved. Several studies have indicated that employing florfenicol in aquaculture can indeed foster the development of AB-resistant bacterial strains [62,64]. To minimize the development of AMR linked to the use of florfenicol in aquaculture, it is imperative to exercise prudent AB administration and adhere to good farming-management practices. This involves utilizing appropriate treatment dosages and durations, reducing the frequency of usage, and enhancing hygiene and disease-management protocols.

*Amoxicillin*. It is a broad-spectrum AB widely used in human medicine to combat bacterial infections, and has also found use in aquaculture for the treatment of bacterial infections in fish and aquatic animals [36]. However, its application in aquaculture has sparked controversy due to concerns about AB resistance and potential environmental and food-chain residues. While amoxicillin is approved for aquaculture use in certain countries, stringent regulations are in place to ensure its safe and responsible application, such as in the EU, USA and Japan [65].

Regardless of the method or purpose of use, AB residues can accumulate in fish tissues before being completely metabolized and eliminated from their bodies [66]. This happens, in particular, when they are given outside the indicated doses or manufacturer’s recommendations. In addition, fish and aquatic organisms found in open waters are not spared from the influence of antimicrobial drugs, due to discharges from farms and other agricultural environments [56,67]. Determining the levels of antimicrobial residues in tissues of aquatic animals can be used to track the source of contamination to which they were exposed [56,68]. In comparison with terrestrial animals, AMU in aquaculture has a greater potential for environmental dissemination, thus exerting a more significant impact on the ecosystem and public health [40,55,69].

### 3.2. AMR Emergence, Transfer and Dissemination in Aquaculture

Antimicrobial-resistant bacteria are widespread in the interconnected ecosystem domains of environment, animals and humans. A profound understanding of the evolution of AMR and the dynamics of resistance dissemination throughout this triad is crucial for anticipating emerging pathogens and managing the spread of AMR [70]. The emergence of AMR can occur through vertical means, involving point mutations, [71] or horizontally, through the diffusion of ARGs and mobile resistance genes, enabling the acquisition of mobile genetic elements like plasmids and transposons and further spreading genetic determinants [62].

AMRs can be categorized into two primary forms: natural (intrinsic) resistance and acquired (transmissible) resistance [72,73]. Natural resistance is an inherent characteristic of a specific bacterial species or genus. It persists as it is transmitted from the parent cell to its progeny, unless subsequent mutations make them vulnerable [74]. It allows microorganisms to withstand the presence of antimicrobial agents due to their innate characteristics [75]. Such inherent resistance is consistent, and it continuously exists within the bacterial species.

In contrast, acquired resistance becomes evident only after exposure to antimicrobial agents. This type of resistance, often referred to as induced resistance, is not an inherent trait of microorganisms but arises as result of their response to external factors [76]. Bacteria can acquire resistance through various mechanisms, such as mutation within chromosomal DNA [72], horizontal gene transfer (HGT) [77,78], or acquiring resistance genes from other bacteria. This property is not consistent or hereditary.

**Figure 2:**
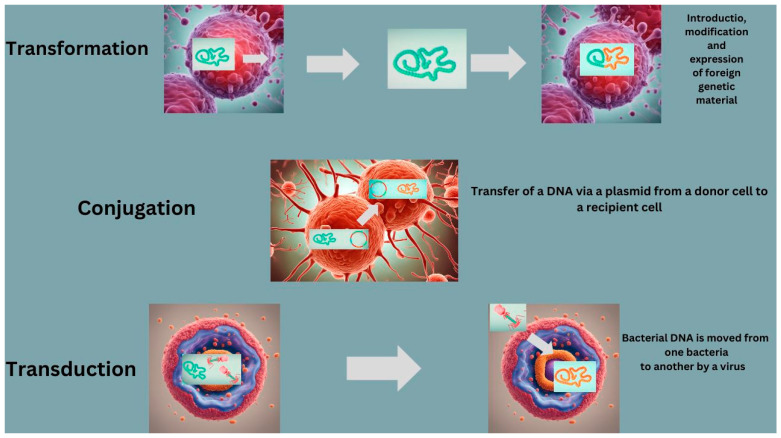
Principal mechanisms for development of AMR via conjugation, transduction and transformation.

There are several ways that AMR can be transferred in aquaculture. For example, it can rapidly disseminate in aquatic bacterial populations through HGT [47,79]. Bacteria resistant to ABs harbor intracellular ARG, which often has the potential to be transmitted to different bacteria via HGT, leading to the emergence of novel AB-resistant bacteria [29]. This can occur through a variety of mechanisms, including conjugation, transduction, and transformation (Figure 2). The transmission mechanisms for ARG between bacterial organisms is elaborated in the further sub-paragraphs.

*Conjugation*. This is the transfer of genetic material between bacteria, through a physical connection. In this process, a plasmid carrying genes for the transfer process (F factor) is transferred from a donor bacterium to a recipient bacterium through a conjugation bridge. The F factor can integrate into the recipient bacterium’s genome and transfer additional genetic material. Conjugation stands out as the primary mechanism responsible for the swift spread of ARGs [80,81].

*Transduction*. It relates to the transfer of genetic material between bacteria through a bacteriophage. During the process of viral replication, the phage can accidentally package bacterial DNA instead of its own DNA, and then transfer this bacterial DNA to another bacterium. This is an efficient pathway for the transfer of ARGs, particularly among bacterial hosts of the same species [82]. The phage-mediated mechanism has been established as a significant driver for the transmission of the ARG related to tetracycline and β-lactam AB in *Staphylococcus aureus*, as documented by some studies [17,83]. Further, transduction is also associated with emergence of multi-drug resistance to variety of Abs, as observed among strains of *Streptococcus pyogenes*, *Enterococci*, *Escherichia coli*, *Salmonella*, and methicillin-resistant *Staphylococcus aureus* (MRSA) [84].

*Transformation*. It is based on uptake of DNA from the environment by a bacterium. Bacteria can take up DNA fragments released by other bacteria that have lysed, or they can take up DNA from the environment [81]. The DNA is then incorporated into the recipient bacterium’s genome through recombination. This is the least-efficient HGT mechanism, primarily because this process exposes DNA to the external environment outside the cell [81].

Evidently, HGT is an important mechanism for bacterial evolution, as it allows bacteria to rapidly acquire new traits such as AB resistance. A number of studies have investigated the possibility that aquaculture systems may represent a reservoir of AMR [85,86].

**Figure 3:**
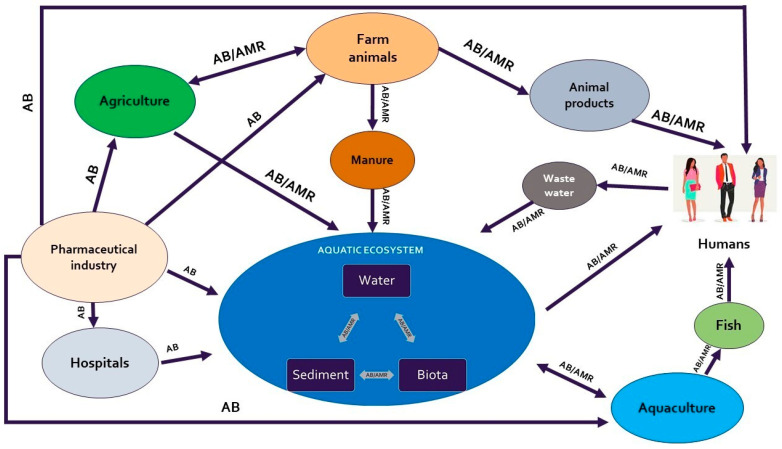
Transmissions routes of antimicrobial agents and AMR in environment.

Further, wastewater treatment plants can be a significant source of AB-resistant bacteria and genes, and effluents from these plants can discharge into aquatic environments. Similarly, agricultural runoff or discharges from livestock facilities can also introduce resistant bacteria and genes into aquatic environments [1]. Overall, industrial waste from pharmaceutical companies and hospitals and runoff from livestock farms and the agri-food sector foster complex interactions between microbiota and the human population that may have great influence on the presence of ARG in aquatic environments (Figure 3) [47,87].

*Contaminated feed*. It is another way in which AMR can be disseminated in the aquatic environment. Aquatic animals, such as fish and shrimp, are often fed with feed that contains ABs or other antimicrobial agents to promote growth or prevent infections. Therefore, use of ABs in animal feed can influence the development of resistant bacteria in the intestines of aquatic animals which become carriers of resistant bacteria and genes [88]. These bacteria can then be released into the environment through the animals’ feces, which can contribute to the dissemination of AMR in the aquatic environment [53]. Further, the existence of antimicrobial-resistant bacteria within wild fish populations can occur via transmission of resistant organisms through water exchange between fish farms and adjacent natural habitats [9,36].

The disposal of unused or expired feed, such as dumping it in water or using it as fertilizer in aquaculture ponds, can also contribute to the dissemination of AMR in the aquatic environment [89]. To reduce the spread of AMR, it is important to promote responsible use of ABs in animal feed, improve feed quality and safety, and implement proper disposal practices for unused or expired feed.

*Antibiotic residues in water and sediment*. The important concern is that AB residues can remain in the water or sediment of aquaculture systems even after the ABs have been removed. These residues can further contribute to the development of AMR. When ABs are used in aquaculture or agriculture, they can leave residues in water bodies through runoff, leaching or discharge of treated effluent. These residues can have negative impacts on the environment, including the selection and spread of AB-resistant bacteria and genes [90]. Further, exposure to AB residues can also induce changes in the microbial community composition and diversity in aquatic environments. This can alter the ecological balance of the microbial community, which can lead to the proliferation of opportunistic pathogens and the suppression of beneficial microorganisms [91].

*Biofilms*. These are other potential hotspots for AMR in aquaculture systems. Consequently, the excessive use of ABs in aquaculture can contribute to the formation of biofilms. Biofilms can form on various surfaces in aquaculture systems, including tanks, nets, and pipes, and can create environments that facilitate the growth and spread of AB-resistant bacteria [49,92,93,94]. The extracellular polymeric substances in the biofilm matrix can form a physical barrier that prevents ABs from reaching the bacteria, making them less susceptible to antimicrobial treatment. Additionally, bacteria in biofilms can exchange genetic material more efficiently, which can increase the likelihood of horizontal transfer of resistance genes [95]. ABs can disrupt the natural microbial communities in aquaculture systems, which can lead to the proliferation of opportunistic pathogens that can form biofilms. Once established, these biofilms can act as a reservoir for AB-resistant bacteria and genes which can persist in the aquaculture system and potentially spread to other bacterial populations. Biofilms can also be difficult to remove, which can contribute to the long-term persistence of AB-resistant bacteria in aquaculture systems, since the use of harsh chemicals to remove biofilms can have negative impacts on the environment and can also promote the selection of AB-resistant bacteria [96,97,98,99].

*Climate change*. Special attention should be given to the climate change impact on the emergence of AMR in aquaculture systems. The rising temperatures may lead to the change in cell physiology of bacteria, thus causing the emergence of AMR. Some aquatoriums worldwide are more susceptible to climate change, such as the Mediterranean Sea [62]. The rising temperatures are a mechanistic modulator that facilitates the transmission of AMR in bacteria, including within aquatic systems [100]. In a study carried out over a five-year period in Europe, it was observed that countries with 10 °C warmer ambience experienced more rapid resistance increases across all AB classes compared to other countries. The reported increase in AMR ranged from 0.33–1.2%/per year, even taking into consideration recognized resistance drivers such as AB consumption and population density. The trends of increased temperatures may foster further global spread of AMR, making risk mitigation strategies more complicated [100].

### 3.3. Clinical Breakpoints and ECV

The data on clinical breakpoints (the concentration of AB used to define whether an infection by a particular bacterial strain is likely to be treatable in a patient) for those resistant bacteria in commercial fisheries are generally scarce. For example, the Minimum Inhibitory Concentration (MIC) (the lowest concentration of an antimicrobial that could prevent visible bacterial growth) and ECV (the highest MIC value of isolates that are not known to have resistance and are therefore considered representative of wild-type bacterial isolates) for *Aeromonas hydrophila* and *Aeromonas veronii* were examined in a study by Woo et al. [12] against several selected ABs; the MICs for these *Aeromonas* spp. isolates ranged from 0.25–64 µg/mL for doxycycline, 0.03–32 µ/mL for enrofloxacin, and 0.03–64 µg/mL for erythromycin and florfenicol, while oxytetracycline had the highest MIC, at >256 µg/mL. The MICs for *Vibrio* spp. were examined in bivalve shellfish by Mancini et al. [101] and it was observed that high resistance percentages with respect to sulfonamide/sulfisoxazole (57.1%; 72/126) (MIC > 256 µg/mL), ampicillin (85.7%; 108/126), and cephalosporins/cefazolin (56.3%; 71/126) were found among all *Vibrio* species. In a study by de Oliveira et al. [102], an MIC assay based on the protocol by Clinical and Laboratory Standards Institute (CLSI, 2014) was used and the provisional ECV for *Streptococcus* (*agalactiae*) in tilapia farming, evaluating the profile of florfenicol resistance, was established, observing that ECV was 8 μg/mL for 94% of the tested strains classified as a wild-type; ECV was calculated using two methodologies: the normalized resistance interpretation (NIR) [103] and ECOFFinder MS (https://clsi.org/meetings/susceptibility-testing-subcommittees/ecoffinder/, accessed on 25 May 2024). The MICs for *Edwardsiella* in aquatic culture was conducted using the disk diffusion test, and provisional ECVs that were established were 8 µg/mL for erythromycin, 10 µg/mL for neomycin, 18 µg/mL for sulfamethoxazole-trimethoprim, 23 µg/mL for amoxicillin, 25 µg/mL for oxytetracycline, 26 µg/mL for norfloxacin, and 27 µg/mL for florfenicol [104]. Further investigation is needed to establish more precisely the ECVs for the aforementioned four most-frequent bacteria associated with AMR in aquaculture.

### 3.4. Detection Methods for AMR

Detecting and monitoring AMR in aquaculture is crucial for gaining a comprehensive overview of potential threats to both animal and human health. The direct correlation between aquatic ecosystems and various human needs, such as water consumption, crop irrigation, and the direct consumption of fish and other aquaculture products, highlights the importance of these activities [84,105]. Additionally, it is essential to monitor the use of antimicrobial agents in human and veterinary medicine within the One Health context encompassing the interface between environment, animals and humans [33].

The determination of AMR profiles in bacterial isolates from aquatic animals often involves various in vitro procedures. These methods go beyond assessing sensitivity to antimicrobial agents and play a crucial role in monitoring the emergence and spread of resistant microorganisms within populations [106]. Ongoing enhancements and applicability of these monitoring techniques are ensured through continuous updates to guidelines and recommendations by relevant organizations, such as the CLSI (USA) and WOAH [107].

Standard classical methods for the detection of bacterial resistance primarily rely on the cultivation of microorganisms under specific conditions. The most-often applied methods include paper diffusion, the disk diffusion method, the Epsilon (E) test based on AB diffusion, [108] broth dilution, agar dilution methods for determining the MIC, [109] and other classical approaches [110]. Although these methods are simple and easy to perform, they have certain limitations that make them less useful. One reason is that certain microorganisms isolated from nature may not be easily cultivable, or their multiplication in the natural environment may take a long time [111]. Additionally, they only provide phenotypic information about bacterial resistance, and cannot detect resistance genes [110,112].

Beyond traditional methods, automated and semi-automated devices based on microdilution susceptibility testing have been widely used in recent decades, providing significantly faster results, such as the VITEK System (bioMérieux, Craponne, France), the Phoenix System (BD Diagnostic Systems, Franklin Lakes, NJ, USA), and the MicroScan Systems (Renton, WA, USA), which stand out as prominent examples [113,114].

Further, modern molecular methods, such as real-time PCR, DNA microarrays or Whole Genome Sequencing (WGS), facilitate the direct determination of genetic determinants responsible for AMR expression. WGS stands as a powerful tool for comprehending the interconnections between the health of aquatic animals, their ecosystems, and human health. The importance of WGS testing is emphasized regarding the occurrence of HGT between the bacteria in these environments and human pathogens, carrying significant public health implications [54,115,116,117]. WGS enables the detection of all the genes responsible for AMR, and thus the possibility of establishing a comprehensive database of all resistance factors within a single species [114]. WOAH guidelines [118] advocate for the integration of genotypic methods with phenotypic analysis to enhance specificity and sensitivity. This is crucial, because the mere presence of ARG does not universally translate to phenotypic resistance.

WGS also facilitates understanding of how ARGs move between different ecosystems, providing invaluable insights within the One Health context for combating AMR effectively. Further, WGS also allows predictions of significant features important for public health, such as resistance to antimicrobials, serotyping, virulence factors, and pathogenicity of isolated strains. It also serves as a guide for conducting monitoring and surveillance and investigating outbreaks, [119] since WGS allows for the tracking of disease transmission pathways among aquatic organisms, helping to identify sources and routes of infection. However, molecular methods (including WGS) for testing AMR include certain drawbacks, such as limitations in detecting only previously recognized resistances, which can lead to false-negative results. Additionally, the inability to define the MIC presents an additional challenge. These methods require validation against phenotypic data and extensive resistance databases, employing innovative bioinformatic approaches. Nevertheless, molecular methods for testing AMR prove to be a safe, efficient, and reliable tool in clinical settings. As experience with these tests grows, along with the collection of data on their effectiveness and clinical impact, they are likely to become more widely accepted [114,120]. Lastly, the routine use of WGS in food safety management was also recommended by the FAO to facilitate the One Health approach. It is expected that it should improve the current knowledge regarding microbiological diversity and genetic information and its application for identification and tracking of microorganisms (including AMR) in food production and food control, and clinical microbiology and epidemiology [121].

### 3.5. Alternatives to Antimicrobial Treatment in Aquaculture

There are several alternatives to the use of antimicrobials in aquaculture. The most important are prebiotics, probiotics, immunostimulants, vaccines, EOs, peptides and phage therapy.

*Prebiotics*. These are non-digestible food ingredients that can stimulate the growth of beneficial bacteria in the gut. thus providing the stable intestinal microbiota and improving growth performance of aquatic animals. The novel functional foods for fish nutrition involves bioactive compounds, including prebiotics, which have beneficial immune responses [122,123]. Non-digestible oligosaccharides promote the growth of intestinal microbiota, enhancing nutrient absorption and bolstering the immune system of fish [124]. There are several prebiotics that are commonly used in aquaculture and can help to stimulate the growth of beneficial bacteria in the intestines and improve the immune system of fish and other aquatic animals, as follows: mannan oligosaccharides derived from the cell walls of yeast [125]; fructo-oligosaccharides derived from fruits and vegetables [126]; galacto-oligosaccharides derived from milk [127]; inulin derived from plants; and chitin and chitosan derived from the shells of crustaceans [128]. For example, the combination of prebiotics in shrimp aquaculture resulted in a significantly higher daily growth rate compared to groups that did not receive them in feed (*p* < 0.05); namely, the highest observed daily growth rate was 8.12% in shrimps that received both prebiotics and probiotics, while the control group had a daily growth rate of 7.18%. The feed-conversion-ratio results showed minimal differences between the experimental groups.

*Probiotics*. These refer to live microorganisms that confer health benefits when consumed in adequate amounts (*Lactic acid bacteria*, *Phaeobacter* spp., *Bacillus* spp.). These microorganisms, predominantly bacteria and yeast, are known for their positive influence on the host organism, typically the human and animal body [122]. Probiotics contribute to the maintenance of a balanced microbial environment, particularly in the digestive system, by promoting a healthy balance of gut bacteria, supporting digestion, and contributing to the modulation of the immune system. The difference in the effect of the use of probiotics in aquaculture compared to the breeding of mammals is that probiotics used in aquaculture can interact with the surrounding environment in a way that leads to an improvement in its quality, and preventing the growth and development of pathogens that could negatively affect the health status of fish [123,129]. The tests on probiotics in aquaculture were mostly directed towards lactic acid bacteria [53,130,131,132]. *Bacillus*, *Vibrio*, *Pseudomonas* and *Aeromona*s were also the subject of research [53,133,134] for their ecology relating to the competitive action of probiotic bacteria.

*Immunostimulants* are substances that can boost the immune system of fish and other aquatic animals. They can help to prevent infections and reduce the need for AB [135]. For example, β-Glucans are complex polysaccharides derived from the cell walls of yeast, bacteria, and fungi [136]. Zinc and selenium are trace minerals that are essential for the proper functioning of the immune system [137]. Vitamin C is an antioxidant that can help to protect fish and other aquatic animals from oxidative stress [138]. They can all help to improve the growth and survival of fish in stressful conditions by activating the immune system of fish and other aquatic animals, leading to an increased production of white blood cells and antibodies. The dosage and administration of immunostimulants may vary depending on the species of fish, the age of the animals, and the specific health conditions present in the aquaculture system.

*Vaccines.* They can be used to prevent bacterial infections in fish and other aquatic animals. At present, bacterial vaccines such as those targeting *A. salmonicida*, *V. anguillarum*, and *Y. ruckeri* are being utilized, with preparations underway for the vaccination against viral diseases [139]. They can be administered through injection or through the feed.

*Essential oils*. Herbs and their extracts are emerging as increasingly promising supplements and alternatives, due to their effectiveness, safety, environmental friendliness, and reduced drug resistance. The effectivenes of herbal medicines in preventing and controlling viral, bacterial, parasitic and fungal fish diseases is due to their robust immune enhancement, anti-oxidation, or direct anti-pathogenic effects of their active components (e.g., polyphenols, polysaccharides, saponins, flavonoids, alkaloids) [140]. A number of studies, mainly on poultry, have proven that the addition of EOs to food leads to a reduction of certain pathogenic microorganisms in the intestines [141,142]. The usage of EOs of *Ocimum gratissimum* and *Hesperozygis ringens* for their antimicrobial and antiparasitic properties, respectively, against different fish pathogens, was studied, and both EOs showed moderate activity against the bacteria *Aeromonas hydrophila* and *Aeromonas veronii* (MIC 400–800 µg/mL) and weak activity against *Citrobacter freundii* and *Raoltella ornithinolytica* [143]. Additionally, some EOs, when used in therapeutic baths at concentrations lower than those needed for sedation, can help prevent oxidative stress in fish. For example, the EO of Melaleuca alternifolia has been shown to prevent the inhibition of splenic creatine kinase and pyruvate kinase activities caused by diseases, indicating its potential as a stress-reducing agent in aquaculture practices [144]. Lippia alba, commonly known as cidreira herb, is a native plant widely found in Brazil. Pharmacological research has demonstrated its analgesic, spasmolytic, and antibacterial properties, with no toxic effects observed in animals. Secondary metabolites in L. alba include flavonoids, tannins, iridoids, triterpenic saponins, resins, mucilage, and essential oil. Terpenoids, particularly mono- and sesquiterpenoids, serve various functions, including protection against oxidative damage and low oxygen levels [145].

*Peptides*. Antimicrobial peptides (AMPs) are short, gene-encoded peptides present in living organisms such as bacteria, insects, plants and vertebrates, as well as humans [146]. AMPs have an important role in the maintenance of microbial ecology and the innate immunity of most organisms [146]. The antimicrobial activity of D-Caerin (synthetic all-D-amino acid peptide) was tested against four *Vibrio* species (*V. aestuarianus*, *V.anguillarum*, *V.harveyi. V.tapetis*), taking into consideration the fact that vibriosis is one of the most usual infection diseases in bivalve mollusks (particularly affecting seed and larvae) and can have a devastating effect in shellfish hatcheries. This is aggravated due to increased resistance of *Vibrio* spp. against traditional AB [147]. It was confirmed that D-Caerin had a much higher antimicrobial action and was significantly more effective than its corresponding natural L-counterpart [147].

*Phage therapy*. By definition, phages (or bacteriophages) are viruses that can infect and kill bacteria [148]. Phages are made of protein shell composed of proteins and nucleic acids [25]. Due to intensification of aquaculture operations worldwide and increased use of antimicrobials (ABs) in prophylactic and therapeutical purposes, thus provoking the emergence of AMR, phage therapy can represent an alternative approach, enabling an effective and sustainable approach to controlling pathogenic bacteria in the aquaculture production chain [148].

By using alternatives to antimicrobials, aquaculture producers can help to reduce the risk of AMR and promote the health of fish and other aquatic animals. It is recommended that strategies for AMR prevention and control should be planned in accordance with internationally recognized frameworks and locally available solutions [139], and these alternatives used to prevent the occurrence of aquatic animals’ illnesses and to reduce the use of antimicrobials.

### 3.6. Risk Mitigation Strategies for AMR in Aquaculture

In addition to what has been previously explained, other synergistic actions should be used in an attempt to enable an integrated approach to combat AMR.

*Innovative Genetic Tools in Aquaculture.* The genetics of farmed fish, by selectively breeding fish for disease resistance, is an alternative solution for disease prevention and for reducing the reliance on ABs [149]. Genomic selection (GS) employs genetic markers spanning the entire genome to compute genomic estimated breeding values for selection candidates [150]. By selecting fish with these markers, farmers can breed for disease-resistant strains. For example, bacterial cold.water disease (BCWD) provoked by *Flavobacterium psychrophilum*, stands as one of the most destructive afflictions in rainbow trout (*Oncorhynchus mykiss*) aquaculture. The utilization of licensed ABs for BCWD treatment amplifies production expenses, with the potential emergence of AB-resistant pathogens. The selective breeding of resistance to BCWD emerges as a viable strategy to address this issue with respect to economic losses, which is aimed at enhancing animal welfare and allowing the production of specific-pathogen-free (SPF) fish (raised in a controlled environment free from specific pathogens) [151].

*Farm hygiene and sanitation management.* Implementation of good agricultural and sanitation practices is a key aspect of AMR prevention and control in aquaculture. For example, the low level of hygienic conditions in the pond environment leads to the bioaccumulation of residues in the form of sediments at the bottom of the pond, which consequently increases the risk of survival of pathogens resistant to antimicrobial drugs [152,153]. When implemented regularly and correctly, hygienic practices can, in turn, reduce the need for AB [154]. This encompasses the application of adequate hygienic conditions in all stages of aquaculture production, from cultivation to processing (e.g., implementation and control of access to farms/pools, disinfection of equipment, facilities, pools and vehicles, control of waste and control of fish health with separation of sick animals).

*Diet*. A proper and balanced diet applied in aquaculture, using a high-quality feed in appropriate quantities, contributes to the prevention of diseases, and indirectly, to reduction of the need for AB [155]. In conditions of excessive feeding and the use of low-quality food, stress and animal diseases occur as a result, which directly affects the increase in the consumption of antimicrobials.

*Disease Prevention and Fish Health Monitoring.* A disease prevention plan can help identify potential disease risks and establish preventive measures, such as vaccination, to reduce the need for AB [28]. Certain vaccines can be used to prevent bacterial infections in fish and other aquatic animals, such as nucleic acid vaccines against *Nocardia seriolae* infection in orange-spotted grouper *Epinephelus coioides* [156] or nano immersion vaccine providing cross-immunoprotection against *Streptococcus agalactiae* and *Streptococcus iniae* infection in tilapia [157].

*Water quality*. Water quality parameters such as temperature, dissolved oxygen, pH, salinity, ammonia, nitrite, and nitrate can affect the health status of the fish in the pond [158]. Namely, poor water quality can increase the risk of disease and stress in aquaculture animals, which opens up the need for the use of ABs. Therefore, effective water quality management is essential to ensure optimal production and profitability of aquaculture operations. For assessment of pond water quality, the following information should be taken into consideration: pond type, pond age, water sources, feed type, pond fertilization, stocking density, and disease incidences [159].

**Figure 4:**
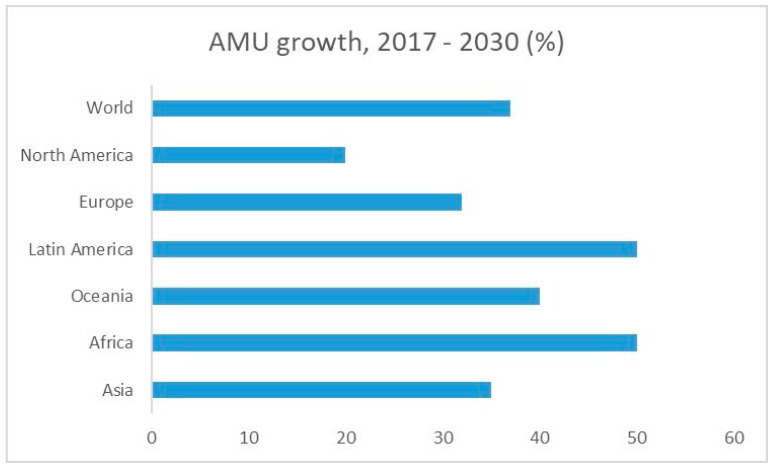
Estimated growth (in %) in AMU in aquaculture globally, between 2017 and 2030 (adapted from Schar et al. [40]).

*AMU and AMR monitoring*. Monitoring of AB use in aquaculture will help to understand the current practices and the associated factors leading to the emergence of AMR [36]. Usually, the misuse/overuse of antimicrobials in fish farming in ponds and/or freshwater systems is due to a lack of training of farmers, poor farmer knowledge on the purposes of AB, and shorter farming experiences [36]. In 2017, global antimicrobial consumption in aquaculture was estimated at 10,259 tons. Projections indicate a 33% increase from this baseline, reaching 13,600 tons by the year 2030. The Asia–Pacific region dominates global consumption, accounting for an overwhelming 93.8%. This proportion is expected to remain constant from 2017 to 2030. Africa (2.3%) and Europe (1.8%) ranked as the second- and third-highest-consuming regions in 2017. Europe’s share is anticipated to decline to 1.7% by 2030, while Africa’s is predicted to rise by 13%, reaching 2.6%. Remarkably, Africa and Latin America demonstrate the most substantial relative increases in consumption, with growth rates of 50.9% and 50.6% (Figure 4), respectively, between 2017 and 2030 [40]. AMU monitoring is related to the collection of data on the antimicrobials taken by animals and humans [160], while AMR monitoring encompasses the prevalence of AB-resistant bacteria in aquaculture. The monitoring of the quantities and usage patterns of antimicrobials in aquatic animals is also recommended in the WOAH Aquatic Animal Health Code [161]. The elements for data collecting are the kilograms of the active ingredient of the antimicrobial agent(s) used per year, divided into antimicrobial class/subclass [161]. The data on AMU can be collected from different sources, such as custom service, import–export, end-use sources (animal business operators), direct sources (wholesalers and feed manufacturers, feed stores and retailers), the pharmaceutical industry, and veterinary associations [120,161].

Tracking the AMR in aquaculture should be conducted within national AMR surveillance and monitoring programs. These programs should be mutually harmonized at regional and/or international level, to provide the same level of public health protection and facilitate global trade. The important component of the national AMR monitoring program is to establish baseline data on the prevalence of antimicrobial-resistant microorganisms and determinants, collect information on AMR trends in relevant microorganisms, and explore the potential relationship between AMR in aquatic animal microorganisms and the use of antimicrobial agents, which are the elements to serve for risk analyses relevant to aquatic animal and human health [161].

It is of particular importance to establish antimicrobial usage thresholds and to impose penalties for non-compliance as integral components of the legislation. The EU Farm-to-Fork Strategy aims to decrease the overall usage of AB in farmed animals and aquaculture across 27 EU Member States, from 118.3 mg/PCU in 2018 to 59.2 mg/PCU by 2030, representing a 50% reduction [162].

Overall, a combination of the aforementioned alternative treatments and preventive measures in a synergistic manner is the best and most effective way to reduce the need for antimicrobials in aquaculture. Regular monitoring of health status of aquatic animals can help identify potential disease outbreaks in the early stage, enabling timely intervention. This includes using ABs only when necessary, using the right AB for the specific disease or infection, and using ABs at the correct dose and duration [163]. Accurate diagnostic testing can help identify the specific pathogens causing disease in fish, which can lead to more targeted and effective use of ABs, thus reducing their use. It is important to work with a veterinarian or other qualified professional to develop a diagnostic testing plan that is appropriate for the farm. For example, such plan should be based on international approaches, such as those of the FAO and the European Inland Fisheries Advisory Commission/EIFAC (https://www.fao.org/fishery/en/organization/eifaac, accessed on 25 May 2024), which have set forth guidelines for monitoring fish health and disease. This includes SPF aquaculture, used when transferring aquatic animals. Information on sample preparation, suitable diagnostic tests, import procedures, and physical-site specifications are given in these guidelines [164,165]. Regular monitoring and record-keeping of AB use (class of AB, dose, duration of treatment) can help identify trends and patterns in use to develop more effective AB stewardship programs.

Between 2000 and 2018, the percentage of antimicrobial compounds with resistance exceeding 50% (P50) in each survey remained steady, at 33% [95% confidence interval (CI) 28 to 37%] in cultured aquatic animals. In contrast, it decreased significantly in wild-caught aquatic animals, dropping from 52% [95% CI 39 to 65%] to 22% [95% CI 14 to 30%] (*p* = 0.003). Over the entire period, the median P50 for surveys of cultured aquatic animals was 31% (n = 558), which was lower than the median P50 for surveys of wild-caught aquatic animals, at 44% (n = 81) (*p* = 0.059) [15].

### 3.7. One Health Approach to Tackle AMR in Aquaculture

**Figure 5:**
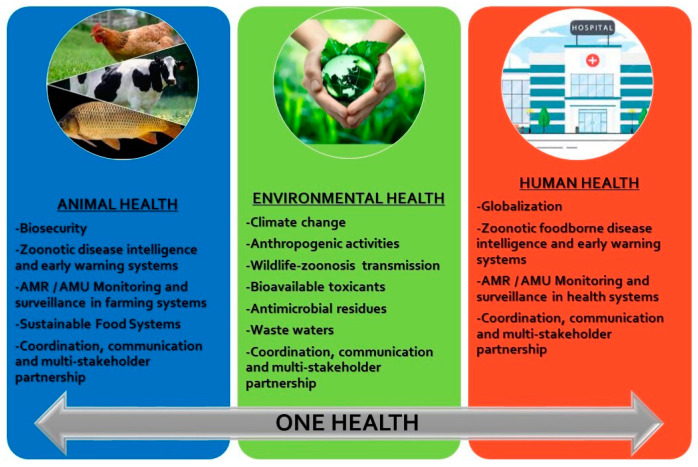
One Health framework: environment–animal–human interface.

The roots of the OH concept can be traced back to ancient civilizations that recognized the link between human health and the environment [166]. The formal recognition and promotion of this holistic approach occurred in the mid-20th century. The concept found its early expression in the fight against zoonotic diseases, such as rabies and brucellosis, where it became evident that addressing health issues in isolation failed to provide comprehensive solutions. As a holistic strategy that recognizes the interconnectedness of environmental, animal and human health, the OH approach became increasingly important in addressing the issue of AMR, particularly in the context of aquaculture (Figure 5). OH interventions in aquaculture to reduce the need for antimicrobials (ABs), include, as follows: (i) alternative disease management strategies, such as use of prebiotics, probiotics, vaccines, peptides and phage therapy; (ii) biosecurity measures to prevent the spread of disease; (iii) surveillance systems to monitor the use of ABs and the emergence of resistance in aquatic environments; and (iv) educating farmers and consumers about AMR risks and the importance of responsible AB use in aquaculture [167].

The Food and Agriculture Organization (FAO) has undertaken efforts to implement risk analysis within the One Health framework, as a vital decision-making tool. Such an approach was especially relevant for the responsible movement of live aquatic animals [168], and has been integrated into the framework of the National Strategy on Aquatic Animal Health [169].

In 2006, a significant milestone in the fight against AMR occurred during the Joint FAO/WOAH/WHO Expert Meeting on AMU and AMR in Aquaculture, recognizing the critical hazards posed by antimicrobial residues and AMR in aquaculture [8]. A subsequent expert workshop held in 2008 identified seven major risk sectors related to aquaculture production [8,169], as follows: (i) pathogen risks, (ii) food safety and public health risks, (iii) ecological (pests) risks, (iv) genetic risks, (v) environmental risks, (vi) financial risks, and (vii) social risks.

The Global Action Plan on AMR, with contributions from the FAO and WOAH, was officially adopted during the 68th World Health Assembly in May 2015 [170]. The World Assembly of WOAH adopted the AMR strategy in May 2015 [171]. In June 2015, the FAO adopted a resolution stating that AMR is an increasingly serious threat to public health and sustainable food production, and that an effective response should involve all sectors of government and society [172].

A critical moment in the fight against AMR came in September 2016, when a high-level meeting on AMR at the 71st United Nations General Assembly (UNGA) resulted in a political declaration [8]. UNGA called for tripartite action, consisting of the FAO as the global leader for food and agriculture, the WOAH as the global leader for animal health and welfare, and the WHO as the global leader for human health, to collaborate with other intergovernmental organizations in supporting the development and implementation of NAP and AMR activities under the One Health platform.

The FAO Action Plan on AMR 2016–2020 was subsequently introduced to support the implementation of Resolution 4/2015. It focuses on four key areas: 1. Raising Awareness (enhancing awareness regarding AMR and its associated threats); 2. Generating Evidence (developing capacity for surveillance and monitoring of AMR and AMU in food and agriculture); 3. Strengthening Governance (enhancing governance related to AMU and AMR in food and agriculture); and 4. Promoting Best Practices (encouraging the adoption of good practices in food and agricultural systems and promoting prudent AMU) [173].

Recently, at the high-level ministerial conference on AMR in Muscat, Oman, the UN supported a quadripartite action to accelerate the combat against AMR within the OH context, by establishing cooperation between the key four international agencies: the FAO, WHO, WOAH and the UN Environment Programme (UNEP) [174,175].

## 4. Conclusions

The widespread use of antimicrobials in aquaculture in some regions of the world, while aiming to prevent and treat bacterial infections in fish, poses a significant public health risk. In comparison with terrestrial animals, AMU in aquaculture has a greater potential for environmental dissemination, thus exerting a more significant impact on the ecosystem and public health. In order to tackle effectively AMR emergence in aquaculture and its spread to the enviroment and food chain, further investigation is needed to establish more precisely the ECVs for most frequent bacteria associated with AMR in fisheries (*Aeromonas* spp., *Vibrio* spp., *Streptococcus agalactiae*, *Edwardsiella*). Modern molecular methods, such as WGS, facilitate the direct determination of genetic determinants responsible for AMR expression. WGS stands as a powerful tool for tracking the interconnections between the health of aquatic animals, their ecosystems, and human health, and the routine use of WGS in food safety management was also recommended by the FAO to facilitate the OH approach. Further, by using alternatives to antimicrobials (prebiotics, probiotics, immunostimulants, vaccines, EOs, peptides, and phage therapy), aquaculture producers can help to reduce the risk of AMR and promote the health of fish and other aquatic animals. Strategies for AMR prevention and control should be in accordance with internationally recognized frameworks and locally available solutions. Risk mitigation strategies against AMR should include innovative genetic tools in aquaculture (GS and the selective breeding of fish for disease resistance), farm hygiene and sanitation management, diet (high-quality feed) and water quality, as well as AMU and AMR monitoring (record-keeping of AMU and class, and duration of treatment) to identify trends and patterns in use. Governments should play a crucial role in combatting AMR in aquaculture through strengthening regulations and enforcement mechanisms based on the EMA, WHO and WOAH categorization of antimicrobials for use in human and veterinary medicine, including establishing antimicrobial usage thresholds and imposing penalties for non-compliance, as integral components of the legislation. Additional efforts should be put into the contribution of the farm environment to AMR development, since this aspect traditionally receives less attention. This is of particular importance for low- and middle-income countries, with the lack of integration of the national AMR NAP with the aquaculture-producing environment. The information on AMR patterns from the pre-harvest (intensive aquaculture/farming) and also post-harvest level (e.g., non-heat-treated ready-to-eat products such as sushi and, cold-smoked salmon) should be included in the NAP. Lastly, the OH approach, connecting environmental, animal and human health, emerges as a vital holistic approach to combat the challenges of AMR in aquaculture. The quadripartite approach to tackle AMR, composed of environmental and animal health, food and agriculture, and public health agencies (the UNEP, WOAH, FAO, and WHO, respectively) provides a good example for the creation of NAPs, since it enables more effective actions to underscore the AMR threat, including in aquaculture. By embracing such an interdisciplinary perspective, a sustainable future for global aquaculture can be envisioned to safeguard both human and animal health, along with environmental well-being.

## Figures and Tables

**Table 1:** Overview of research on AMR in aquaculture.

Author(s)	Year	Study Location	Microorganism(s)	Type of Samples	Antimicrobial Agent(s)	Phase in Aquaculture Production Chain
**Raza et al.** [42]	2022	South Korea	/	Water samples	Sulfonamides ^VCIA,HIA,D^ Tetracyclines ^VCIA,HIA,D^ Quinolones ^VCIA,HPCIA,B^ Beta lactams ^VCIA,HIA,D^	Pre-harvest
**Girijan et al.** [43]	2020	India	*Escherichia coli*	Sediment, water, fish/shrimp/clams	Ciprofloxacin ^VCIA,HPCIA,B ^ Levofloxacin ^VCIA,HPCIA,B^ Moxifloxacin ^VCIA,HPCIA,B^ Ofloxacin ^VCIA,HPCIA,B^ Norfloxacin ^VCIA,HPCIA,B^ Nalidixic acid ^VCIA,HPCIA,B^ Gentamicin ^VCIA,CIA,C^ Amikacin ^VCIA,CIA,C^ Cefotaxime ^VCIA,HPCIA,B^ Cefotetan ^HIA,C^ Ceftazidime ^VCIA,HPCIA,B^ * Imipenem ^VCIA,D^ Colistin ^VCIA,HPCIA,B^	
**Yang et al.** [44]	2023	China	*Proteobacteria*, Firmicutes, *Enterococcus* spp., *Escherichia* spp., *Streptococcus* spp., *Klebsiella* spp., *Acinetobacter* spp., *Bacteroidetes*, *Cyanobacteria*, *Klebsiella pneumoniae*,	Farm worker feces, water, sediment, fish guts, duck manure	Aminoglycosides ^VCIA,CIA,C^ Phenicoles ^VCIA,HIA,C^ Tetracyclines ^VCIA,HIA,D^ Sulfonamides ^VCIA,HIA,D^	Pre-harvest
**Bell et al.** [45]	2023	Bangladesh	/	Water samples	Aminoglycosides ^VCIA,CIA,C^ Sulphonamides ^VCIA,HIA,D^ * Carbapenems ^VCIA,A^ Cephalosporins ^VCIA,HPCIA/HIA,B^ Cephamycins ^VCIA,HIA,D^ Penams ^VCIA,CIA,D^ Fluoroquinolones ^VCIA,HPCIA,B^ Diaminopyrimidines ^VCIA,CIA,D^ Phenicols ^VCIA,HIA^	N/A
**Jones et al.** [46]	2023	57 countries	*Aeromonas* spp.	Human, wastewater, drinking water, surface water, agriculture	Aminoglycosides ^VCIA,CIA,C^ * Carbapenems ^VCIA,A^ Cephalosporins ^VCIA,HPCIA/HIA,B^ Fluoroquinolones ^VCIA,HPCIA,B^ Macrolides ^VCIA,CIA,C^ * Monobactams ^A^ Penicillins ^VCIA,HIA^ Phenicols ^VCIA,HIA^ Polypeptides ^VHIA^ Sulfonamides ^VCIA,HIA,D^ Tetracyclines ^VCIA,HIA,D^	N/A
**Kampouris et al.** [47]	2022	/	*Flavobacterium*, *Pseudomonas*, *Lactococcus*, *Sphingomonas*	Water samples, biofilm from plastic mechanical filters from ponds with African catfish (*Clarias gariepinus*)	Sulfonamides ^VCIA,HIA,D^ Beta lactams ^VCIA,HIA,D^ Quinolones ^VCIA,HPCIA,B^ Macrolides ^VCIA,CIA,C^	Pre-harvest
**Garza et al.** [35]	2022	Global/ review paper	General	General	General	N/A
**Reddy et al.** [48]	2022	Global/ review paper	General	General	General	N/A
**Brunton et al.** [26]	2019	Vietnam	/	Striped catfish (*Pangasianodon* *hypophthalmus*), White-leg shrimp (*Penaeus vannamei*)	N/A	Pre-harvest
**Chowdhury et al.** [36]	2022	Bangladesh	/	Various fish species	Oxytetracycline ^VCIA,HIA,D^ Ciprofloxacin ^VCIA,HPCIA,C^ Amoxicillin ^VCIA,HIA,D^ Levofloxacin ^VCIA,HPCIA,B^ Erythromycin ^VCIA,CIA,C^ Sulfadiazine ^VCIA,HIA,D^ Trimethoprim ^VCIA,HIA,D^	N/A
**Osman et al.** [49]	2021	Egypt	*Pseudomonas* spp.	Nile tilapia (*Oreochromis niloticus*)	Sulphamethoxazole ^HIA,D^ Trimethoprim ^VCIA,HIA,D^ Amikacin ^VCIA,CIA,C^ * Imipenem ^VCIA,D^ Tetracyclines ^VCIA,HIA,D^ Ampicillin ^VCIA,HIA,D^ Nalidixic acid ^VCIA,HPCIA,B^ Chloramphenicol ^VCIA,HIA,C ^ Gentamicin ^VCIA,CIA,C^ Ciprofloxacin ^VCIA,HPCIA,C^ * Aztreonam ^A^ Ampicillin/Sulbactam ^VCIA,HIA,D^ Cefepime ^VCIA,HPCIA,D ^ Ceftriaxone ^VCIA,HPCIA,B^ Cephalotin ^VCIA,CIA,B^ Cefotaxime ^VCIA,HPCIA,B^ Ceftazidime ^VCIA,HPCIA,B^	Post-harvest
**Algammal et al.** [14]	2022	Egypt	*Edwardsiella tarda*	Nile tilapia (*Oreochromis niloticus*), African catfish (*Clarias gariepinus*)	Amoxicillin ^VCIA,HIA,D^ Ampicillin ^VCIA,HIA,D^ Cefotaxime ^VCIA,HPCIA,B^ Erythromycin ^VCIA,CIA,C^ Streptomycin ^VCIA,CIA,C^ Gentamycin ^VCIA,CIA,C^ Enrofloxacin ^VCIA,HPCIA,B^ Ciprofloxacin ^VCIA,HPCIA,B^ Colistin-sulfate ^VHIA,HPCIA,B^ Tetracycline ^VCIA,HIA,D^ Trimethoprim ^VCIA,HIA,D^ Sulfamethoxazole ^VCIA,HIA,D^	Pre-harvest
**Alhaji** [9]	2021	Nigeria	/	African catfish (*Clarias gariepinus*, *Clarias lazera*)	Tetracyclines ^VCIA,HIA,D^ Penicillin ^VCIA,HIA^ Sulfonamides ^VCIA,HIA,D^ Streptomycin ^VCIA,CIA,C^ Neomycin ^VCIA,CIA,C^ Ampicillin ^VCIA,HIA,D^ Colistin ^VCIA,HPCIA,B^ Erythromycin ^VCIA,CIA,C^ Enrofloxacin ^VCIA,HPCIA,B^ Chloramphenicol ^VCIA,HIA,C^	Pre-harvest

World Organization for Animal Health (WOAH/OIE) categorisation: VCIA—Veterinary Critically Important Antimicrobials; VHIA—Veterinary, Highly Important Antimicrobials [50]. WHO categorisation: HPCIA—Highest Priority Critically Important Antimicrobials; CIA—Critically Important Antimicrobials; HIA—Highly Important Antimicrobials; *—Authorized for use in humans only [51]. EMA categorisation: A—Category A (Avoid); B—Category B (Restrict); C—Category C (Caution); D—Category D (Prudence) [52]. Pre-harvest: aquaculture farming system; post-harvest: aquatic animals after the completion of the rearing and growth cycle.

## Data Availability

The data presented in this study are available on request from the corresponding author. The data are not publicly available due to privacy restrictions.

## List of Abbreviations

**Abreviation**	**Definition**
AB	Antibiotic
AMU	Antimicrobial Use
AMPs	Antimicrobial Peptides
AMR	Antimicrobial Resistance
ARG	Antimicrobial Resistance Gene
BCWD	Bacterial Cold-Water Disease
CIA	Critically Important Antimicrobials
CLSI	Clinical and Laboratory Standards Institute
DALYs	Disability-Adjusted Life Years
ECV	Epidemiological Cut-Off Value
EEA	European Economic Area
EOs	Essential Oils
EU	European Union
FAO	Food and Agriculture Organization
GS	Genomic Selection
HGT	Horizontal Gene Transfer
MIC	Minimum Inhibitory Concentration
NAP	National Action Plan
SPF	Specific-Pathogen-Free
VCIA	Veterinary Critically Important Antimicrobials
WGS	Whole Genome Sequencing
WHO	World Health Organization
WOAH	World Organisation for Animal Health

## References

[1] Iwu C.D., Korsten L., Okoh A. (2020). The incidence of antibiotic resistance within and beyond the agricultural ecosystem: A concern for public health. Microbiol. Open.

[2] WHO (2023). Antimicrobial Resistance. https://www.who.int/news-room/fact-sheets/detail/antimicrobial-resistance.

[3] Zdolec N., Veskovic-Moracanin S., Filipovic I., Dobranic V., Zdolec N. (2016). Antimicrobial Resistance of Lactic Acid Bacteria in Fermented Meat Products. Fermented Meat Products: Health Aspects.

[4] Murray C.J.L., Ikuta K.S., Sharara F., Swetschinski L., Aguilar G.R., Gray A., Han C., Bisignano C., Rao P., Wool E. (2022). Global burden of bacterial antimicrobial resistance in 2019: A systematic analysis. Lancet.

[5] OECD-European Commission Directorate-General for Health and Food Safety (2017). A European One Health Action Plan against Antimicrobial Resistance (AMR). https://health.ec.europa.eu/system/files/2020-01/amr_2017_action-plan_0.pdf.

[6] OECD/WHO (2022). Addressing the Burden of Infections and Antimicrobial Resistance Associated with Health Care. https://www.oecd.org/health/Addressing-burden-of-infections-and-AMR-associated-with-health-care.pdf.

[7] Jasovský D., Littmann J., Zorzet A., Cars O. (2016). Antimicrobial resistance-a threat to the world’s sustainable development. Ups. J. Med. Sci..

[8] FAO (2018). Report of the FAO Expert Working Group Meeting “Scoping Exercise to Increase the Understanding of Risks of Antimicrobial Resistance (AMR) in Aquaculture. Palermo, Italy. Report No.: FIAA/R1268 (En). https://www.fao.org/3/ca7442en/CA7442EN.pdf.

[9] Alhaji N.B., Maikai B.V., Kwaga J. (2021). Antimicrobial use, residue and resistance dissemination in freshwater fish farms of north-central Nigeria: One health implications. Food Control.

[10] World Bank (2017). Drug-Resistant Infections: A Threat to Our Economic Future.

[11] Deng Y., Xu L., Chen H., Liu S., Guo Z., Cheng C., Ma H., Feng J. (2020). Prevalence, virulence genes, and antimicrobial resistance of *Vibrio* species isolated from diseased marine fish in South China. Sci. Rep..

[12] Woo S.J., Kim M.S., Jeong M.G., Do M.Y., Hwang S.D., Kim W.J. (2022). Establishment of Epidemiological Cut-Off Values and the Distribution of Resistance Genes in *Aeromonas hydrophila* and *Aeromonas veronii* Isolated from Aquatic Animals. Antibiotics.

[13] Oviedo-Bolaños K., Rodríguez-Rodríguez J.A., Sancho-Blanco C., Barquero-Chanto J.E., Pena-Navarro N., Escobedo-Bonilla C.M., Umana-Castro R. (2021). Molecular identification of *Streptococcus* sp. and antibiotic resistance genes present in Tilapia farms (*Oreochromis niloticus*) from the Northern Pacific region, Costa Rica. Aquac. Int..

[14] Algammal A.M., Mabrok M., Ezzat M., Alfifi K.J., Esawy A.M., Elmasry N., El-Tarabili R.M. (2022). Prevalence, antimicrobial resistance (AMR) pattern, virulence determinant and AMR genes of emerging multi-drug resistant *Edwardsiella tarda* in Nile tilapia and African catfish. Aquaculture.

[15] Schar D., Zhao C., Wang Y., Larsson D.G.J., Gilbert M., van Boeckel T.P. (2021). Twenty-year trends in antimicrobial resistance from aquaculture and fisheries in Asia. Nat. Commun..

[16] Caputo A., Bondad-Reantaso M.G., Karunasagar I., Hao B., Gaunt P., Verner-Jeffreys D., Fridman S., Dorado-Garcia A. (2022). Antimicrobial resistance in aquaculture: A global analysis of literature and national action plans. Rev. Aquac..

[17] Bobate S., Mahalle S., Dafale N.A., Bajaj A. (2023). Emergence of environmental antibiotic resistance: Mechanism, monitoring and management. Environ. Adv..

[18] EU (2005). Ban on Antibiotics as Growth Promoters in Animal Feed Enters into Effect. Report No.:IP/05/1687. https://ec.europa.eu/commission/presscorner/detail/en/IP_05_1687.

[19] EU (2019). Regulation (EU) 2019/6 of the European Parliament and of the Council of 11 December 2018 on Veterinary Medicinal Products and Repealing Directive 2001/82/EC. https://eur-lex.europa.eu/legal-content/EN/TXT/PDF/?uri=CELEX:32019R0006.

[20] Kirchnelle C. (2018). Swann Song. British antibiotic regulation in livestock production (1953–2006). Bull. Hist. Med..

[21] Lees P., Pelligand L., Giraud E., Toutain P.-L. (2021). A history of antimicrobial drugs in animals: Evolution and revolution. J. Vet. Pharmacol. Ther..

[22] Klein E., van Boeckel T.P., Martinez E.M., Laxminarayan R. (2018). Global increase and geographic convergence in antibiotic consumption between 2000 and 2015. Proc. Natl. Acad. Sci. USA.

[23] Thornber K., Verner-Jeffreys V., Hincliffe S., Rahman M.M., Bass D., Tyler C.R. (2020). Evaluating antimicrobial resistance in the global shrimp industry. Rev. Aquac..

[24] Van Boeckel T.P., Brower C., Gilbert M., Grenfel B.T., Levin S.A., Robinson T.P., Teillant A., Laxminarayan R. (2015). Global trends in antimicrobial use in food animals. Proc. Natl. Acad. Sci. USA.

[25] Sharma A., Singh A., Dar M.A., Kaur R.J., Charan J., Iskandar K., Haque M., Murti K., Ravichandiran V., Dhingra S. (2022). Menace of antimicrobial resistance in LMICs: Current surveillance practices and control measures to tackle hostility. J. Infect. Public Health.

[26] Brunton L.A., Desbois A.P., Garza M., Wieland B., Mohan C.V., Häsler B., Tam C.C., Le P.N.T., Phuong N.T., Van P.T. (2019). Identifying hotspots for antibiotic resistance emergence and selection, and elucidating pathways to human exposure: Application of a systems thinking approach to aquaculture systems. Sci. Total Environ..

[27] Henriksson P., Rico A., Troell M., Klinger D., Buschmann A., Saksida S., Chadag M., Zhang W. (2018). Unpacking factors influencing antimicrobial use in global aquaculture and their implication for management: A review from a systems perspective. Sustain. Sci..

[28] Santos L., Ramos F. (2018). Antimicrobial resistance in aquaculture: Current knowledge and alternatives to tackle the problem. Int. J. Antimicrob. Agents.

[29] Williams-Nguyen J., Sallach J.B., Bartelt-Hunt S., Boxall A.B., Durso L.M., McLain J.E., Singer R.S., Snow D., Zilles J.L. (2016). Antibiotics and Antibiotic Resistance in Agroecosystems: State of the Science. J. Environ. Qual..

[30] Government of Canada Canadian Integrated Program for Antimicrobial Resistance Surveillance (CIPARS) 2006 Guelph. In Public Health Agency of Canada. 2009. CIPARS_annualReport_e.indd (publications.gc.ca). Canadian Integrated Program for Antimicrobial Resistance Surveillance (CIPARS)—Canada.ca. https://www.canada.ca/en/public-health/services/surveillance/canadian-integrated-program-antimicrobial-resistance-surveillance-cipars.html.

[31] Kim J.J., Seong H.J., Johnson T.A., Cha C.J., Sul W.J., Chae J.C. (2023). Persistence of antibiotic resistance from animal agricultural effluents to surface water revealed by genome-centric metagenomics. J. Hazard. Mater..

[32] He Y., Yuan Q., Mathieu J., Stadler L.B., Senehi N.L., Sun R., Alvarez P.J.J. (2020). Antibiotic resistance genes from livestock waste: Occurrence, dissemination, and treatment. NPJ Clean Water.

[33] Nastasijevic I., Proscia F., Jurica K., Veskovic-Moracanin S. (2023). Tracking Antimicrobial Resistance Along the Meat Chain: One Health Context. Food Rev. Int..

[34] Tiseo K., Huber L., Gilbert M., Robinson T.P., van Boeckel T.P. (2020). Global Trends in Antimicrobial Use in Food Animals from 2017 to 2030. Antibiotics.

[35] Garza M., Mohan C.V., Brunton L., Wieland B., Hasler B. (2022). Typology of interventions for antimicrobial use and antimicrobial resistance in aquaculture systems in low- and middle-income countries. Int. J. Antimicrob. Agents.

[36] Chowdhury S., Rheman S., Debnath N., Delamare-Deboutteville J., Akhtar Z., Ghosh S., Parveen S., Islam K., Islam A., Rashid M. (2022). Antibiotics usage practices in aquaculture in Bangladesh and their associated factors. One Health.

[37] Cabello F.C., Godfrey H.P., Buschmann A.H., Dölz H.J. (2016). Aquaculture as yet another environmental gateway to the development and globalisation of antimicrobial resistance. Lancet Infect. Dis..

[38] Cabello F.C., Godfrey H.P., Tomova A., Ivanova L., Dölz H., Millanao A., Buschmann A.H. (2013). Antimicrobial use in aquaculture re-examined: Its relevance to antimicrobial resistance and to animal and human health. Environ. Microbiol..

[39] Miller R.A., Harbottle H. (2018). Antimicrobial Drug Resistance in Fish Pathogens. Microbiol. Spectr..

[40] Schar D., Klein E.Y., Laxminarayan R., Gilbert M., Van Boeckel T. (2020). Global trends in antimicrobial use in aquaculture. Sci. Rep..

[41] Reed T.A.N., Krang S., Miliya T., Townell N., Letchford J., Bun S., Sar B., Osbjer K., Seng S., Chou M. (2019). Antimicrobial resistance in Cambodia: A review. Int. J. Infect. Dis..

[42] Raza S., Choi S., Lee M., Shin J., Son H., Wang J., Kim Y.M. (2022). Spatial and temporal effects of fish feed on antibiotic resistance in coastal aquaculture farms. Environ. Res..

[43] Girijan S.K., Paul R., Kumar V.J.R., Pillai D. (2020). Investigating the impact of hospital antibiotic usage on aquatic environment and aquaculture systems: A molecular study of quinolone resistance in *Escherichia coli*. Sci. Total Environ..

[44] Yang J.-T., Xiao D.-Y., Zhang L.-J., Chen H.-X., Zheng X.-R., Xu X.-L., Jiang H.-X. (2023). Antimicrobial resistome during the transition from an integrated to a monoculture aquaculture farm in southern China. Sci. Total Environ..

[45] Bell A.G., Thornber K., Chaput D.L., Hasan N.A., Alam M.M., Haque M.M., Cable J., Temperton B., Tyler C.R. (2023). Metagenomic assessment of the diversity and ubiquity of antimicrobial resistance genes in Bangladeshi aquaculture ponds. Aquac. Rep..

[46] Jones D.C., LaMartina E.L., Lewis J.R., Dahl A.J., Nadig N., Szabo A., Newton R.J., Skwor T.A. (2023). One Health and Global Health View of Antimicrobial Susceptibility through the “Eye” of *Aeromonas*: Systematic Review and Meta-Analysis. Int. J. Antimicrob. Agents.

[47] Kampouris I., Alygizakis N., Klümper U., Agrawal S., Lackner S., Cacace D., Kunze S., Thomaidis N., Slobdonik J., Berendonk T. (2022). Elevated levels of antibiotic resistance in groundwater during treated wastewater irrigation associated with infiltration and accumulation of antibiotic residues. J. Hazard. Mater..

[48] Reddy S., Kaur K., Barathe P., Shriram V., Govarthanan M., Kumar V. (2022). Antimicrobial resistance in urban river ecosystems. Microbiol. Res..

[49] Osman K.M., da Silva Pires A., Franco O.L., Saad A., Hamed M., Naim H., Ali A.H.M., Elbehiry A. (2021). Nile tilapia (*Oreochromis niloticus*) as an aquatic vector for *Pseudomonas* species of medical importance: Antibiotic Resistance Association with Biofilm Formation, Quorum Sensing and Virulence. Aquaculture.

[50] WOAH (2021). OIE List of Antimicrobials of Veterinary Importance. https://www.woah.org/app/uploads/2021/03/oie-list-antimicrobials.pdf.

[51] WHO (2024). WHO List of Medically Important Antimicrobials. A Risk Management Tool for Mitigating Antimicrobial Resistance Due to Non-Human Use. https://cdn.who.int/media/docs/default-source/gcp/who-mia-list-2024-lv.pdf?sfvrsn=3320dd3d_2.

[52] EMA (2019). Categorisation of Antibiotics in the European Union. Answer to the Request from the European Commission for Updating the Scientific Advice on the Impact on Public Health and Animal Health of the Use of Antibiotics in Animals. https://www.ema.europa.eu/en/documents/report/categorisation-antibiotics-european-union-answer-request-european-commission-updating-scientific-advice-impact-public-health-and-animal-health-use-antibiotics-animals_en.pdf.

[53] Romero J., Feijoo C., Navarrete P., Carvalho E. (2012). Antibiotics in Aquaculture—Use, Abuse and Alternatives. Health and Environment in Aquaculture.

[54] Liu X., Steele J.C., Meng X.Z. (2017). Usage, residue, and human health risk of antibiotics in Chinese aquaculture: A review. Environ. Pollut..

[55] Lulijwa R., Rupia E.J., Alfaro A. (2019). Antibiotic use in aquaculture, policies and regulation, health and environmental risks: A review of the top 15 major producers. Rev. Aquac..

[56] Carrizo J.C., Griboff J., Bonansea R.I., Nimptsch J., Valdes M.E., Wunderlin D.A., Ame M.V. (2021). Different antibiotic profiles in wild and farmed Chilean salmonids. Which is the maun source for antibiotic in fish?. Sci. Total Environ..

[57] Zhang Q.Z., Li X.M. (2007). Pharmacokinetics and residue elimination of 1108 oxytetracycline in grass carp, *Ctenopharyngodon idellus*. Aquaculture.

[58] Leal J.F., Santos E.B.H., Esteves V.I. (2019). Oxytetracycline in intensive aquaculture: Water quality during and after its administration, environmental fate, toxicity and bacterial resistance. Rev. Aquac..

[59] Navarrete P., Mardones P., Opazo R., Espejo R., Romero J. (2008). Oxytetracycline treatment reduces bacterial diversity of intestinal microbiota of Atlantic salmon. J. Aquat. Anim. Health.

[60] Smith P., Samuelsen O.B. (1996). Estimates, of the significance of out-washing of oxytetracycline from sediments under Atlantic salmon sea-cages. Aquaculture.

[61] Almeida S.A.A., Heitor A.M., Montenegro M.C.B.S.M., Sales M.G.F. (2011). Sulfadiazine-selective determination in aquaculture environment: Selective potentiometric transduction by neutral or charged ionophores. Talanta.

[62] Pepi M., Focardi S. (2021). Antibiotic-Resistant Bacteria in Aquaculture and Climate Change: A Challenge for Health in the Mediterranean Area. Int. J. Environ. Res. Public Health.

[63] Zhang Q., Li N., Wang B.-L., Ma P.-F., Zhang W.-X., Nusrat Z.S., Ma Z., Zhang Y.-Q., Ying L. (2022). Capabilities and mechanisms of microalgae on nutrients and florfenicol removing from marine aquaculture wastewater. J. Environ. Manag..

[64] Hektoen H., Berge J.A., Hormazabal V., Yndestad M. (1995). Persistence of antibacterial agents in marine sediments. Aquaculture.

[65] Stephen J., Mukherjee S., Lekshmi M., Kumar S.H., Mothadaka M.P., Vaiyapuri M., Rao Badireddy M., Nagarajrao Ravishankar C., Bhatia R., Jena J. (2023). Diseases and Antimicrobial Use in Aquaculture. Handbook on Antimicrobial Resistance.

[66] Okocha R.C., Olatoye I.O., Adedeji O.B. (2018). Food safety impacts of antimicrobial use and their residues in aquaculture. Public Health Rev..

[67] Varol M., Rasit M. (2019). Environmental contaminants in fish species from a large dam reservoir and their potential risks to human health. Ecotoxicol. Environ. Saf..

[68] Harwood J.J. (2014). Molecular markers for identifying municipal, domestic and agricultural sources of organic matter in natural waters. Chemosphere.

[69] Boxall A.B.A., Fogg L.A., Blackwell P.A., Kay P., Pemberton E.J., Croxford A. (2004). Veterinary medicines in the environment. Rev. Environ. Contam. Toxicol..

[70] Robinson T.P., Bu D.P., Carrique-Mas J., Fèvre E.M., Gilbert M., Grace D., Hay S.I., Jiwakanon J., Kakkar M., Kariuki S. (2016). Antibiotic resistance is the quintessential One Health issue. Trans. R. Soc. Trop. Med. Hyg..

[71] Yelin I., Kishony R. (2018). Antibiotic Resistance. Cell.

[72] Martinez J.L. (2014). General principles of antibiotic resistance in bacteria. Drug Discov. Today Technol..

[73] Arzanlou M., Chai W.C., Venter H. (2017). Intrinsic, adaptive and acquired antimicrobial resistance in gram-negative bacteria. Essays Biochem..

[74] Reygaert W.C. (2018). An overview of the antimicrobial resistance mechanisms of bacteria. AIMS Microbiol..

[75] Blair J.M.A., Webber M.A., Baylay A.J., Ogbolu D.O., Piddock L.J.V. (2015). Molecular mechanisms of antibiotic resistance. Nat. Rev. Microbiol..

[76] Cox G., Wright G.D. (2013). Intrinsic antibiotic resistance: Mechanisms, origins, challenges and solutions. Int. J. Med. Microbiol..

[77] Coculescu B.I. (2009). Antimicrobial resistance induced by genetic changes. J. Med. Life..

[78] Davies J., Davies D. (2010). Origins and Evolution of Antibiotic Resistance. Microbiol. Mol. Biol. Rev..

[79] Berendonk T., Manaia C., Merlin C., Fatta-Kassinos D., Cytryn E., Walsh F., Bürgmann H., Sørum H., Norström M., Pons M.N. (2015). Tackling antibiotic resistance: The environmental framework. Nat. Rev. Microbiol..

[80] Lerner A., Matthias T., Aminov R. (2017). Potential effects of horizontal gene exchange in the human gut. Front. Immunol..

[81] McInnes R.S., McCallum G.E., Lamberte L.E., van Schaik W. (2020). Horizontal transfer of antibiotic resistance genes in the human gut microbiome. Curr. Opin. Microbiol..

[82] Lerminiaux N.A., Cameron A.D.S. (2019). Horizontal transfer of antibiotic resistance genes in clinical environments. Can. J. Microbiol..

[83] Peterson E., Kaur P. (2018). Antibiotic resistance mechanisms in bacteria: Relationships between resistance determinants of antibiotic producers, environmental bacteria, and clinical pathogens. Front. Microbiol..

[84] von Wintersdorff C.J., Penders J., van Niekerk J.M., Mills N.D., Majumder S., van Alphen L.B., Savelkoul P.H., Wolffs P.F. (2016). Dissemination of Antimicrobial Resistance in Microbial Ecosystems through Horizontal Gene Transfer. Front. Microbiol..

[85] Muziasari W., Pärnänen K., Johnson T., Lyra C., Karkman A., Stedtfeld R., Tamminen M., Tiedje J., Virta M. (2016). Aquaculture changes the profile of antibiotic resistance and mobile genetic element associated genes in Baltic Sea sediments. FEMS Microbiol. Ecol..

[86] Reverter M., Sarter S., Caruso D., Avarre J.C., Combe M., Pepey E., Pouyaud L., Vega-Heredía S., de Verdal H., Gozlan R. (2020). Aquaculture at the crossroads of global warming and antimicrobial resistance. Nat. Commun..

[87] Bielen A., Šimatović A., Kosić-Vukšić J., Senta I., Ahel M., Babić S., Jurina T., Plaza J.J.G., Milaković M., Udiković-Kolić N. (2017). Negative environmental impacts of antibiotic-contaminated effluents from pharmaceutical industries. Water Res..

[88] Hasan J., Rahman H., Ullah R., Mredul M.H. (2020). Availability of aqua drugs and their uses in semi intensive culture farms at Patuakhali district in Bangladesh. Arch. Agric. Environ. Sci..

[89] Suyamud B., Chen Y., Quyen D.T.T., Dong Z., Zhao C., Hu J. (2024). Antimicrobial resistance in aquaculture: Occurrence and strategies in Southeast Asia. Sci. Total Environ..

[90] Vilca F.Z., Galarza N.C., Tejedo J.R., Cuba W.A.Z., Quiróz C.N.C., Tornisielo V.L. (2021). Occurrence of residues of veterinary antibiotics in water, sediment and trout tissue (*Oncorhynchus mykiss*) in the southern area of Lake Titicaca, Peru. J. Gt. Lakes Res..

[91] Chen J., Li W., Zhang J., Qi W., Li Y., Chen S., Zhou W. (2020). Prevalence of antibiotic resistance genes in drinking water and biofilms: The correlation with the microbial community and opportunistic pathogens. Chemosphere.

[92] Lécuyer F., Bourassa J.S., Gélinas M., Charron-Lamoureux V., Burrus V., Beauregard P. (2018). Biofilm Formation Drives Transfer of the Conjugative Element ICEBs1 in *Bacillus subtilis*. Appl. Environ. Sci..

[93] Abe K., Nomura N., Suzuki S. (2020). Biofilms: Hot spots of horizontal gene transfer (HGT) in aquatic environments, with a focus on a new HGT mechanism. FEMS Microbiol. Ecol..

[94] Ge Z., Ma Z., Zou J., Zhang Y., Li Y., Zhang L., Zhang J. (2023). Purification of aquaculture wastewater by macrophytes and biofilm systems: Efficient removal of trace antibiotics and enrichment of antibiotic resistance genes. Sci. Total Environ..

[95] Kampouris I.D., Klümper U., Kramer L., Sorum H., Wedekind H., Berendonk T.U. (2022). Dissemination of antibiotic resistance in antibiotic-free recirculating aquaculture systems. J. Hazard. Mater. Adv..

[96] Casciaro B., Dutta D., Loffredo M.R., Marcheggiani S., McDermott A.M., Willcox M.D., Mangoni M.L. (2018). Esculentin-1a derived peptides kill *Pseudomonas aeruginosa* biofilm on soft contact lenses and retain antibacterial activity upon immobilization to the lens surface. Pept. Sci..

[97] Crouzet M., Claverol S., Lomenech A.M., Le Senechal C., Costaglioli P., Barthe C., Garbay B., Bonneu M., Vilain S. (2017). *Pseudomonas aeruginosa* cells attached to a surface display a typical proteome early as 20 min of incubation. PLoS ONE.

[98] Oliveira W.F., Silva P.M.S., Silva R.C.S., Silva M.M., Machado G., Coelho L., Correia M.T.S. (2018). *Staphylococcus aureus* and *Staphylococcus epidermidis* infections on implants. J. Hosp. Infect..

[99] Cappiello F., Loffredo M.R., Del Plato C., Cammarone S., Casciaro B., Quaglio D., Mangoni M.L., Botta B., Ghirga F. (2020). The Revaluation of Plant-Derived Terpenes to Fight Antibiotic-Resistant Infections. Antibiotics.

[100] McGough S.F., MacFadden D.R., Hattab M.W., Mølbak K., Santillana M. (2020). Rates of increase of antibiotic resistance and ambient temperature in Europe: Across-national analysis of 28 countries between 2000 and 2016. Eurosurveillance.

[101] Mancini M.E., Alessiani A., Donatiello A., Didonna A., D’Attoli L., Faleo S., Occhiochiuso G., Carella F., Di Taranto P., Pace L. (2023). Systematic Survey of *Vibrio* spp. and *Salmonella* spp. in Bivalve Shellfish in Apulia Region (Italy): Prevalence and Antimicrobial Resistance. Microorganisms.

[102] de Oliveira T.F., Queiroz G.A., Teixeira J.P., Figueiredo H.C.P., Leal C.A.G. (2018). Recurrent *Streptoccoccus agalactiae* infection in Nile tilapia (*Oreochromis niloticus*) treated with florfenicol. Aquaculture.

[103] Kronvall G. (2010). Normalized resistance interpretation as a tool for establishing epidemiological MIC susceptibility breakpoints. J. Clin. Microbiol..

[104] Reis F.Y.T., Rocha V.P., Janampa-Sarmiento P.C., Costa H.L., Egger R.C., Passos N.C., de Assis C.H.S., Carneiro S.P., Santos F., Silva B.A. (2023). *Edwardsiella tarda* in Tambaqui (*Colossoma macropomum*): A Pathogenicity, Antimicrobial Susceptibility, and Genetic Analysis of Brazilian Isolates. Animals.

[105] Bengtsson-Palme J., Kristiansson E., Larsson D.G.J. (2018). Environmental factors influencing the development and spread of antibiotic resistance. FEMS Microbiol. Rev..

[106] Jorgensen J.H., Turnidge J.D., Jorgensen J.H., Carroll K.C., Funke G., Pfaller M.A., Landry M.L., Richter S.S., Warnock D.W., Richter S.S., Patel J.B. (2015). Susceptibility Test Methods: Dilution and Disk Diffusion Methods. Manual of Clinical Microbiology.

[107] Yalew S.T. (2020). Review on Antibiotic Resistance: Resistance Mechanisms, Methods of Detection and Its Controlling Strategies. Biomed. J. Sci. Tech. Res..

[108] Aksentijević K., Ašanin J., Nišavić J., Marković M., Milanov D., Mišić D. (2017). Antibiotic resistance in bacteria isolated from fish in Serbia. Vet. Glas..

[109] Michel C., Blanc G. (2001). Minimal inhibitory concentration methodology in aquaculture: The temperature effect. Aquaculture.

[110] Jiang G.L., Yu S.Y., Yu W.Q., Zhou X., Zhou Z.M., Li H. (2022). Research progress in the detection techniques and methods of bacterial drug resistance and drug-resistant genes. J. Chin. Clin. Med..

[111] Galhano B.S.P., Ferrari R.G., Panzenhagen P., de Jesus A.C.S., Conte-Junior C.A. (2021). Antimicrobial Resistance Gene Detection Methods for Bacteria in Animal-Based Foods: A Brief Review of Highlights and Advantages. Microorganisms.

[112] Gao Z., Piao Y., Hu B., Yang C., Zhang X., Zheng Q., Cao J. (2023). Investigation of antibiotic resistance genotypic and phenotypic characteristics of marine aquaculture fish carried in the Dalian area of China. Front. Microbiol..

[113] Benkova M., Soukup O., Marek J. (2020). Antimicrobial susceptibility testing: Currently used methods and devices and the near future in clinical practice. J. Appl. Microbiol..

[114] Gajic I., Kabic J., Kekic D., Jovicevic M., Milenkovic M., Mitic Culafic D., Trudic A., Ranin L., Opavski N. (2022). Antimicrobial Susceptibility Testing: A Comprehensive Review of Currently Used Methods. Antibiotics.

[115] Martinez J.L. (2009). The role of natural environments in the evolution of resistance traits in pathogenic bacteria. Proc. R. Soc. B-Biol. Sci..

[116] Aedo S., Ivanova L., Tomova A., Cabello F.C. (2014). Plasmid-related quinolone resistance determinants in epidemic *Vibrio parahaemolyticus*, uropathogenic *Escherichia coli*, and marine Bacteria from an aquaculture area in Chile. Microb. Ecol..

[117] Rajan V., Sivaraman G.K., Vijayan A., Elangovan R., Prendiville A., Bachmann T.T. (2022). Genotypes and phenotypes of methicillin-resistant staphylococci isolated from shrimp aquaculture farms. Environ. Microbiol. Rep..

[118] OIE Laboratory methodologies for bacterial antimicrobial susceptibility testing. In OIE Terrestrial Manual; World Organisation for Animal Health. 2012. fmd with Viaa Test Incl (woah.org). https://www.woah.org/fileadmin/Home/eng/Health_standards/tahm/2.01.01_ANTIMICROBIAL.pdf.

[119] Uelze L., Grützke J., Borowiak M., Hammerl J.A., Juraschek K., Deneke C., Tausch S.H., Malorny B. (2020). Typing methods based on whole genome sequencing data. One Health Outlook..

[120] Devadas S., Zakaria Z., Shariff M., Bhassu S., Karim M., Natrah I. (2024). Methodologies and standards for monitoring antimicrobial use and antimicrobial resistance in shrimp aquaculture. Aquaculture.

[121] FAO (2016). Technical Meeting on the Impact of Whole Genome Sequencing on Food Safety Management within a One Health Approach. https://www.fao.org/3/i6582e/i6582e.pdf.

[122] Manning T.S., Gibson G.R. (2004). Prebiotics. Best Pract. Res. Clin. Gastroenterol..

[123] Bondad-Reantaso M.G., MacKinnon B., Karunasagar I., Fridman S., Alday-Sanz V., Brun E., Le Groumellec M., Li A., Surachetpong W., Karunasagar I. (2023). Review of alternatives to antibiotic use in aquaculture. Rev. Aquac..

[124] Oviedo-Olvera M.V., Feregrino-Pérez A.A., Nieto-Ramírez M.I., Tovar-Ramírez M.M., Aguirre-Becerra H., García-Trejo J.F. (2023). Forthcoming. Prebiotic emergent sources for aquaculture: Microalgae and insects. Aquac. Fish.

[125] Merrifield D.L., Dimitroglou A., Foey A., Davies S.J., Baker R.T.M., Bøgwald J., Castex M., Ringø E. (2010). The current status and future focus of probiotic and prebiotic applications for salmonids. Aquaculture.

[126] Kumar C.G., Sripada S., Poornachandra Y., Grumezescu M., Holban A.M. (2018). Chapter 14—Status and Future Prospects of Fructooligosaccharides as Nutraceuticals. Handbook of Food Bioengineering, Role of Materials Science in Food Bioengineering.

[127] Muzaffar K., Jan R., Bhat N.A., Gani A., Shagoo M.A., Dhanasekaran D., Sankaranarayanan A. (2021). Chapter 25—Commercially Available Probiotics and Prebiotics Used in Human and Animal Nutrition. Advances in Probiotics.

[128] Oktaviana A., Widanarni Yuhana M. (2014). The Use of Synbiotics to Prevent IMNV and *Vibrio harveyi* Co-Infection in *Litopenaeus vannamei*. HAYATI J. Biosci..

[129] Banerjee G., Ray A.K. (2017). The advancement of probiotics research and it application in fish farming industries. Res. Vet. Sci..

[130] Olmos J., Ochoa L., Paniagua-Michael J., Contreras L. (2011). Functional feed assessment on *Litopenaeus vannamei* using 100% fish meal replacement by soybean meal, high levels of complex carbohydrates and *Bacillus* probiotic strains. Mar. Drugs.

[131] Antony S., Singh I.S.B., Jose R.M., Kumar P.R.A., Philip R. (2011). Antimicrobial peptide gene expression in tiger shrimp, *Penaeus monodon* in response to gram-positive bacterial probionts and white spot virus challenge. Aquaculture.

[132] Sun Y., Yang H.L., Ma R.L., Lin W.Y. (2010). Probiotic applications of two dominant gut *Bacillus* strains with antagonistic activity improved the growth performance and immune responses of grouper *Epinephelus coioides*. Fish Shellfish Immunol..

[133] Tovar-Ramírez D., Mazurais D., Gatesoupe J., Patrick Q. (2010). Dietary probiotic live yeast modulates antioxidant enzyme activities and gene expression of sea bass (*Dicentrarchus labrax*) larvae. Aquaculture.

[134] Abdeltawwab M., Abdelrahman A., Ismael N. (2008). Evaluation of commercial live bakers’ yeast, *Saccharomyces cerevisiae* as a growth and immunity promoter for Fry Nile tilapia, *Oreochromis niloticus* (L.) challenged in situ with *Aeromonas hydrophila*. Aquaculture.

[135] Vijayaram S., Ringø E., Zuorro A., van Doan H., Sun Y. (2024). Forthcoming. Beneficial roles of nutrients as immunostimulants in aquaculture: A review. Aquac. Fish.

[136] Bragg R.R., Meyburgh C.M., Lee J.Y., Coetzee M., Adhikari R., Thapa S. (2018). Potential treatmentoptions in a post-antibiotic era. Infectious Diseases and Nanomedicine.

[137] Vijayaram S., Sun Y.Z., Zuorro A., Ghafarifarsani H., van Doan H., Hoseinifar S.H. (2022). Bioactive immunostimulants as health-promoting feed additives in aquaculture: A review. Fish Shellfish Immunol..

[138] El Basuini M.F., Shahin S.A., Teiba I.I., Zaki M.A., El-Hais A.M., Sewilam H., Almeer R., Abdelkhalek N., Dawood M.A.O. (2021). The influence of dietary coenzyme Q10 and vitamin C on the growth rate, immunity, oxidative-related genes, and the resistance against Streptococcus agalactiae of Nile tilapia (*Oreochromis niloticus*). Aquaculture.

[139] Senthamarai M.D., Rajan M.R., Bharathi P.V. (2023). Current risks of microbial infections in fish and their prevention methods: A review. Microb. Pathog..

[140] Zhang W., Zhao J., Ma Y., Li J., Chen X. (2022). The effective components of herbal medicines used for prevention and control of fish diseases. Fish Shellfish Immunol..

[141] Jang I.S., Ko Y.H., Kang S.Y., Lee C.Y. (2007). Effect of a commercial essential oil on growth performance, digestive enzyme activity and intestinal microflora population in broiler chickens. Anim. Feed Sci. Technol..

[142] Cross D., McDevitt R.M., Hillman K., Acamovic T. (2007). The effect of herbs and their associated essential oils on performance, dietary digestibility and gut microflora in chickens from 7 to 28 days of age. Br. Poult. Sci..

[143] Bandeira G., Pês T.S., Saccol E.M.H., Sutili F.J., Rossi W., Murari A.L., Heinzmann B.M., Pavanato M.A., de Vargas A.C., de L. Silva L. (2017). Potential uses of *Ocimum gratissimum* and *Hesperozygis ringens* essential oils in aquaculture. Ind. Crops Prod..

[144] Souza C.F., Baldissera M.D., Baldisserotto B., Heinzmann B.M., Martos-Sitcha J.A., Mancera J.M. (2019). Essential Oils as Stress-Reducing Agents for Fish Aquaculture: A Review. Front. Physiol..

[145] Azambuja C.R., Mattiazzi J., Riffel A.P.K., Finamor J.A., Garcia L.O., Heldwein C.G., Heinzmann B.M., Baldisserotto B., Pavanato M.A., Llesuy S.F. (2011). Effect of the essential oil of *Lippia alba* on oxidative stress parameters in silver catfish (*Rhamdia quelen*) subjected to transport. Aquaculture.

[146] Lazzaro B.P., Zasloff M., Rolf J. (2022). Antimicrobial peptides: Application informed by evolution. Science.

[147] López-Sanmartín M., Rengel R., Lopez-Lopez M., Lebron J.A., Molina-Marquez A., de la Rosa I., Lopez-Cornejo P., Cuesta A., Vigara J., Leon R. (2023). D-amino acid peptides as antimicrobial agents against vibrio-associated diseases in aquaculture. Aquaculture.

[148] Culot A., Grosset N., Gautier M. (2019). Overcoming the challenges of phage therapy for industrial aquaculture: A review. Aquaculture.

[149] Fjalestad K.T., Gjedrem T., Gjerde B. (1993). Genetic improvement of disease resistance in fish: An overview. Aquaculture.

[150] Meuwissen T.H.E., Hayes B.J., Goddard M.E. (2001). Prediction of Total Genetic Value Using Genome-Wide Dense Marker Maps. Genetics.

[151] Vallejo R.L., Evenhuis J.P., Cheng H., Fragomeni B.O., Gao G., Liu S., Long R.L., Shewbridge K.L., Silva R.M.O., Wiens G.D. (2022). Genome-wide mapping of quantitative trait loci that can be used in marker-assisted selection for resistance to bacterial cold water disease in two commercial rainbow trout breeding populations. Aquaculture.

[152] Baquero F., Martinez J.L., Canton R. (2008). Antibiotics and antibiotic resistance in water environments. Curr. Opin. Biotechnol..

[153] Pruden A., Larsson D.G., Amezquita A., Collignon P., Brandt K.K., Graham D.W., Lazorcha J.M., Zhu Y. (2013). Management options for reducing the release of antibiotics and antibiotic resistance genes to the environment. Environ. Health Perspect..

[154] Moges F., Endris M., Belyhun Y., Worku W. (2014). Isolation and characterization of multiple drug resistance bacterial pathogens from waste water in hospital and non-hospital environments, Northwest Ethiopia. BMC Res. Notes.

[155] Lall S.P., Cruz-Suárez L.E., Ricque-Marie D., Tapia-Salazar M., Olvera-Novoa M.A., Civera-Cerecedo R. (2000). Nutrition and health of fish. Avances en Nutrición Acuícola, V. Memorias del V Simposium Internacional de Nutrición Acuícola.

[156] Nguyen P.T.D., Giovanni A., Maekawa S., Pham T.H., Wang P.C., Chen S.C. (2023). An Integrated in silico and in vivo study of nucleic acid vaccine against *Nocardia seriolae* infection in orange-spotted grouper *Epinephelus coioides*. Fish Shellfish Immunol..

[157] Cao Y., Liu J., Liu G., Du H., Liu T., Liu T., Li P., Yu Q., Wang G., Wang E. (2024). A nanocarrier immersion vaccine encoding surface immunogenic protein confers cross-immunoprotection against *Streptococcus agalactiae* and *Streptococcus iniae* infection in tilapia. Fish Shellfish Immunol..

[158] Buttner J.K., Soderberg R.W., Terlizzi D.E. (1993). An Introduction to Water Chemistry in Freshwater Aquaculture. (Publication No. 170-1993).

[159] Mramba R.P., Kahindi E.J. (2023). Pond water quality and its relation to fish yield and disease occurrence in small-scale aquaculture in arid areas. Heliyon.

[160] McNulty K., Soon J.M., Wallace C.A., Nastasijevic I. (2016). Antimicrobial resistance monitoring and surveillance in the meat chain: A report from five countries in the European Union and European Economic Area. Trends Food Sci. Technol..

[161] WOAH (2022). Aquatic Animal Health Code. Twenty-Fourth Edition. https://rr-africa.woah.org/wp-content/uploads/2022/09/en_csaa-2022.pdf.

[162] ESVAC (2022). Sales of Veterinary Antimicrobial Agents in 31 European Countries in 2022. Trends from 2010 to 2022. Thirteenth ESVAC Report. https://www.ema.europa.eu/en/documents/report/sales-veterinary-antimicrobial-agents-31-european-countries-2022-trends-2010-2022-thirteenth-esvac-report_en.pdf.

[163] Kawsar A., Alam T., Pandit D., Rahman M., Mia M., Talukdar A., Sumon T.A. (2022). Status of disease prevalence, drugs and antibiotics usage in pond-based aquaculture at Narsingdi district, Bangladesh: A major public health concern and strategic appraisal for mitigation. Heliyon.

[164] Turner G.E., FAO (1989). Codes of Practice and Manual of Procedures for Consideration of Introductions and Transfers of Marine and Freshwater Organisms.

[165] Murray K.N., Clark T.S., Kebus M.J., Kent M.L. (2022). Specific Pathogen Free—A review of strategies in agriculture, aquaculture, and laboratory mammals and how they inform new recommendations for laboratory zebrafish. Res. Vet. Sci..

[166] Lee E. (2019). Environmental Health Perspectives in the Ancient World. Senior Honors Thesis.

[167] Wei L., Su Z., Yue Q., Huang X., Wei M., Wang J. (2024). Microplastics, heavy metals, antibiotics, and antibiotic resistance genes in recirculating aquaculture systems. TrAC Trends Anal. Chem..

[168] FAO/NACA (2000). Asia Regional Technical Guidelines on Health Management for the Responsible Movement of Live Aquatic Animals and the Beijing Consensus and Implementation Strategy. Report No.: FAO Fisheries Technical Paper No. 402. https://www.fao.org/3/x8485e/x8485e.pdf.

[169] FAO (2008). Understanding and Applying Risk Analysis in Aquaculture. FAO Fisheries and Aquaculture Technical Paper 519. https://www.fao.org/3/i1136e/i1136e00.htm.

[170] WHO (2015). 68th World Health Assembly: WHA Resolution 68.7. Geneva, Switzerland. Report No.: WHA68/2015/REC/1. https://apps.who.int/gb/ebwha/pdf_files/WHA68-REC1/A68_R1_REC1-en.pdf.

[171] OIE (2016). Combating Antimicrobial Resistance through a One Health Approach: Actions and OIE Strategy. OIE General Session. Report No.: 36. https://www.woah.org/fileadmin/Home/eng/Our_scientific_expertise/docs/pdf/AMR/A_RESO_AMR_2016.pdf.

[172] FAO CL 145/Report Food and Agriculture Organization 6. 6. 2015—And Consultative Preparatory Process of the Resolution and Adopted the following Resolution: Resolution 4/2015. Antimicrobial Resistance. Thirty-Ninth Session, Rome, 6–13 June 2015. Report No.: C 2015/REP. https://www.fao.org/3/mo153e/mo153e.pdf.

[173] FAO (2016). The FAO Action Plan on Antimicrobial Resistance 2016–2020. https://www.fao.org/3/i5996e/i5996e.pdf.

[174] UN (2022). The Report of the Third High-Level Ministerial Conference on Antimicrobial Resistance. https://amrconference2022.om/index.

[175] WHO (2022). Quadripartite Launches a New Platform to Tackle Antimicrobial Resistance Threat to Human and Animal Health and Ecosystems. https://www.who.int/news/item/18-11-2022-quadripartite-launches-a-new-platform-to-tackle-antimicrobial-resistance-threat-to-human-and-animal-health-and-ecosystems.

